# ﻿The genus *Argopistes* Motschulsky from Japan and Taiwan, with descriptions of three new species from Taiwan (Coleoptera, Chrysomelidae, Galerucinae, Alticini)

**DOI:** 10.3897/zookeys.1215.134871

**Published:** 2024-10-15

**Authors:** Chi-Feng Lee, Ming-Yao Chiang, Haruki Suenaga

**Affiliations:** 1 Applied Zoology Division, Taiwan Agricultural Research Institute, Taichung 413, Taiwan Applied Zoology Division, Taiwan Agricultural Research Institute Taichung Taiwan; 2 Nakashima, 108-11, Kurashiki-shi, Okayama, 710-0803 Japan Unaffiliated Okayama Japan

**Keywords:** *Chionanthus*, *Fraxinus*, *Jasminum*, *Ligustrum*, *Olea*, Oleaceae, *Osmanthus*, *Syringa*

## Abstract

Four previously described species of *Argopistes* are recognized and redescribed from Japan and Taiwan: *A.biplagiatus* Motschulsky, 1860, *A.rufus* Chen, 1934, *A.tsekooni* Chen, 1934, and *A.unicolor* Jacoby, 1885. Three new species from Taiwan, *A.jungchani***sp. nov.**, *A.tsoui***sp. nov.**, and *A.yuae***sp. nov.**, are described. Descriptions of species include illustrations of aedeagi, antennae, gonocoxae, abdominal ventrite VIII, and spermathecae. *Argopistesrufus* Chen, 1934, **stat. nov.** is raised to species status from a variety of *A.biplagiatus* Motschulsky, 1860. *Argopistescoccinelliformis* Csiki, 1940, **syn. nov.** and *A.ryukyuensis* Shigetoh & Suenaga, 2022, **syn. nov.** are proposed as junior synonyms of *A.rufus* Chen, 1934 Lectotypes are designated for *A.undecimmaculata* Jacoby, 1885, *A.unicolor* Jacoby, 1885, and A.biplagiatusvar.rufus Chen, 1934.

## ﻿Introduction

The flea beetle genus *Argopistes* Motschulsky, 1860 contains 44 species recorded from Afrotropical, Australian, Neotropical, Oriental, and Palearctic regions (Blanco and Konstantinov 2013; Biondi et al. 2024). Four species were known from Japan and reviewed by Kimoto (1965) with emphasis on male aedeagi. A new species was also described from Ryukyu Islands and Daitô Islands (Shigetoh and Suenaga 2022). Chûjô (1936) was the first to record the genus from Taiwan as *A.biplagiatus* Mostschulsky, 1860, although Gressitt and Kimoto (1963) indicated that it was a misidentification of *A.coccinelliformis* Csiki, 1940. No other records have been reported from Taiwan since then.

Adults and larvae of *Argopistes* are oligophagous on Oleaceae (Jolivet and Hawkeswood 1995). A number of species of Oleaceae are ornamental trees popular in Japan, including Osmanthus×fortunei Carrière, *O.heterophyllus* (G. Don) P. S. Green, and *Ligustrumjaponicum* Thunb. Although few insect pests are reported for these ornamental trees, *A.rufus* Chen, 1934 and *A.biplagiatus* Motschulsky, 1860 are major pests. Ecology of both species have been studied in this context (Inoue and Shinkaji 1989a–c, 1990; Inoue 1990a, b, 1991a, b, 1992, 1993, 1994, 1996, 1998, 2001, 2014). In contrast, Chinese privet, *Ligustrumsinense* Lour., is one of the worst invasive plants in the U.S. *Argopistestsekooni* Chen, 1934 was evaluated as a promising biological control agent of Chinese privet (Zhang et al. 2008a, b, 2009).

**Figure 1.**
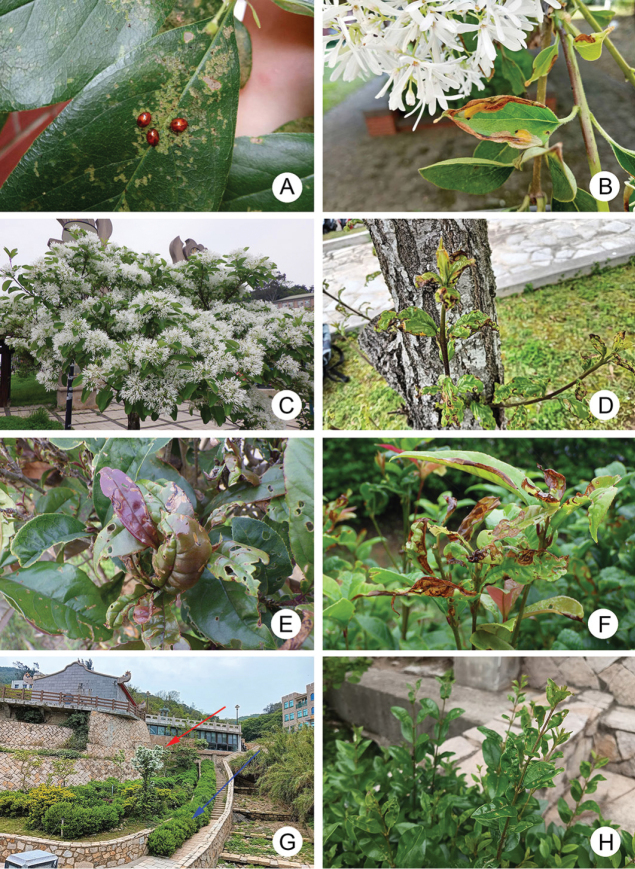
Field photographs of *Argopistesrufus* Chen **A** adults feeding on leaves of *Chionanthusretusus* surrounding Hsinchu City Government on April 23, 2021 **B** mature larvae mining leaves of the same tree **C** blooming *C.retusus* at Qingshui Village (清水村), Nangan Island (南竿島), on April 21, 2024 **D** larvae mining leaves near the ground of the same tree **E** larvae mining leaves of *Osmanthusfragrans* at Yingshan Temple (鶯山廟), Kinmen Island (金門島), on April 11, 2023 **F** larvae mining leaves of *Osmanthusfragrans* at the guesthouse, Jinhu Township (金湖鎮), Kinmen Island, on May 20, 2024 **G***C.retusus* (red arrow) and *Ligustrumjaponicum* (blue arrow) planting surrounding Chinbe Village (芹壁村), Beigan Island (北竿島) on 22 April 22 2024 **H** feeding marks caused by adults on leaves of *L.japonicum*.

In Taiwan, *Chionanthusretusus* Lindley & Paxton (流蘇) (Fig. 1A–D, G), Chinese fringetree, and *Osmanthusfragrans* (Thunb.) Lour. (桂花), sweet osmanthus, are popular ornamental plants. They have been attacked by *Argopistes* species during recent years. This phenomenon also occurs on small islands, including Kinmen Island (Fig. 1E, F), Nangan Island (Fig. 1C, D), and Beigan Island (Fig. 1G, H). Taxonomic studies on *Argopistes* in Taiwan and Japan are needed to describe diagnostic characters in addition to male aedeagi.

## ﻿Materials and methods

For taxonomic study, abdomens of adults were separated from the forebodies and boiled in 10% KOH solution, followed by washing in distilled water to prepare genitalia for illustrations. The genitalia were then dissected from the abdomens, mounted on slides in glycerin, and studied and drawn using a Leica M165 stereomicroscope. For detailed examinations, a Nikon ECLIPSE 50i microscope was used.

At least three males and females from each species were examined to delimit variability of diagnostic characters. For species collected from more than one locality or with color variations, at least one pair of each sex from each locality and color morph was examined. Length was measured from the anterior margin of the eye to the elytral apex, and width at the greatest width of the elytra. Nomenclature for morphological structures of adults follows Duckett and Daza (2004). Names of plant species follows the Taiwan Encyclopedia of Life (2024; TaiEOL).

Specimens studied herein are deposited at the following institutes and collections:

**HAPC** Private Collection of Haruki Suenaga, Okayama, Japan;

**HIPC** Private Collection of Hiroaki Shigetoh, Sapporo, Japan;

**IZAS**Institute of Zoology, Chinese Academy of Sciences, Beijing, China [Yongying Ruan];

**NHMUK**The Natural History Museum, London, UK [Michael F. Geiser, Maxwell V. L. Barclay];

**SEHU**The Laboratory for Systematic Entomology, Hokkaido University, Sapporo, Japan [Takuya Takemoto];

**TAFI**Forest Arthropod Collection of Taiwan, Taiwan Forestry Research Institute, Taipei City, Taiwan [Sheng-Shan Lu];

**TARI**Applied Zoology Division, Taiwan Agricultural Research Institute, Taichung, Taiwan [Chi-Feng Lee];

**ZMMU**Zoological Museum of Moscow State University, Moscow, Russia [Vladimir Savitsky].

Exact label data are cited for all type specimens of described species; a double slash (//) divides the data on different labels and a single slash (/) divides the data in different rows. Other comments and remarks are in square brackets: [p] – preceding data are printed, [h] – preceding data are handwritten, [w] – white label, [y] – yellow label, [g] – green label, [b] – blue label, and [r] – red label. Traditional Chinese fonts are added to the names of localities.

## ﻿Taxonomic account

### ﻿ Argopistes biplagiatus

Taxon classificationAnimaliaColeopteraChrysomelidae

Motschulsky, 1860

1E6FA533-3D44-50B2-82C0-1DE5C5B9AB4E

Figs 2A–F , 3 , 4

Argopistes biplagiatus Motschulsky, 1860: 236 (Amur: Russian Far East and northeastern China); Csiki 1940: 523 (catalogue); Chûjô and Kimoto 1961: 174 (catalogue); Kimoto 1965: 436 (redescription); Lee and An 2001: 182 (South Korea); Lee and Cho 2006: 91 (host plants); Takizаwa 2012: 38 (faunistics).
Argopistes flavitarsis Motschulsky, 1860: 137 (chromatic variation).
Argopistes limbatus Motschulsky, 1860: 137 (chromatic variation).
Argopistes suturalis Motschulsky, 1860: 137 (chromatic variation).
Argopistes undecimmaculata Jacoby, 1885: 738 (Japan: Sapporo); Chûjô 1936: 109 (catalogue); Csiki 1940: 524 (catalogue).

#### Type material examined.

**Figure 2.**
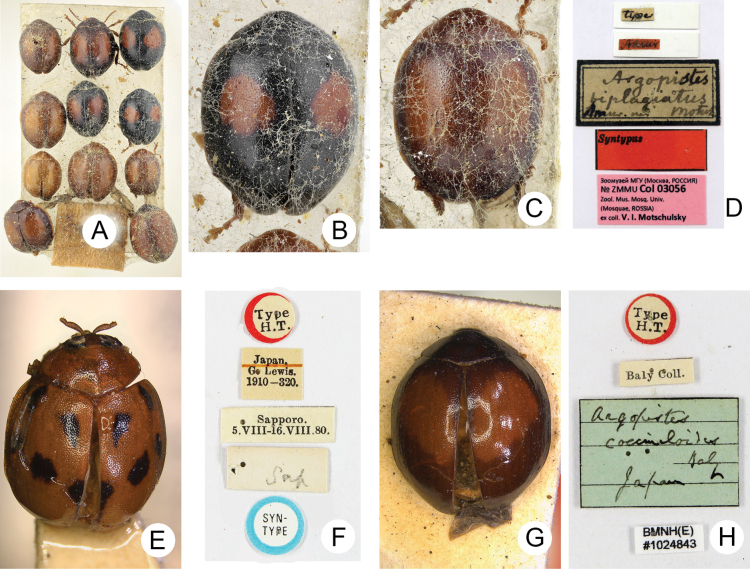
Type specimens and labels **A***Argopistesbiplagiatus* Motschulsky, 1860, syntypes **B** one syntype with typical color form **C** one syntype with enlarged red spots on elytra **D** labels pinned with syntypes **E***A.undecimmaculata* Jacoby, 1885, lectotype **F** labels pinned with lectotype **G***A.coccinelloides* Baly, 1874, holotype **H** labels pinned with holotype.

*Argopistesbiplagiatus*. • 11 ***syntypes*** glued on the same card (ZMMU) (Fig. 2A–D): “type [h, w] // Amur [h, r] // Argopistes / biplagiatus / Amur. m. Motsch [h, w, with black border] // Syntypus [p, r] // Ȝoomyȝeň Mry (Mockba, POCCNR) / No ZMMU Col 03056 / Zool. Mus. Mosq. Univ. / (Mosquae, RUSSIA) / ex coll. V. I. Motschulsky [p, pink label]”.

*Argopistesundecimmaculata*. ***Lectotype*** • (here designated, sex undetermined, NHMUK) (Fig. 2E, F): “Type / H.T. [p, w, circle label with red border] // SYN- / TYPE [p, w, circle label with blue border] // Sapporo / 5.VIII-16.VIII.80. [p, w] // Japan / G. Lewis. / 1910-320 [p, w] // Sap [h, w]”. ***Paralectotypes*** • 1 (sex undetermined, NHMUK): “SYN- / TYPE [p, circle label with blue border] // Sapporo / 5.VIII-16.VIII.80. [p, w] // Japan/ G. Lewis. / 1910-320 [p, w] // Argopistes / 11maculata Jac [h, b]”; • 1♀ (TARI): “Sapporo [h] / JAPAN [p] / 10.VIII.1880 [h] / Col. G. LEWIS [p, w] // Argopistes / undecimmaculata / Jacoby [h] / DET. M. CHUJO [p, w] // CO / Type [p, w, circle label with yellow letters and border] / 1526 [p, w]”.

#### Additional material examined.

**Japan.** Hokkaido: • 1♀ (HAPC), Sapporo-shi, Hokkaido University, 15.X.2011, leg. H. Suenaga; Honshu. Aichi: • 1♂ (SEHU), Toyohashi-shi, Imou-shitsugen, 8.IV.1989, leg. Y. Komiya; Ibaraki: • 1♀ (HIPC), Daigo, Uenomiya, Mt. Yamizo-san, 28.V.2917, leg. H. Yoshitake; • 1♂ (SEHU), Sakura-mura, Sakura-gawa Riv., 1.VI.1986, leg. Y. Komiya; Ishikawa: • 1♀ (HAPC), Mt. Haku-san, Betsuzan-dô, 21.V.2016, leg. H. Kawase; Shizuoka: • 1♂, 2♀ (SEHU), Izu-peninsula, Mt. Manzaburo-dake, 19.V.1980, leg. J. Okuma; • 1♀ (SEHU), Tagata-gum, Tohi, 4.V.1985, leg. Y. Komiya; Tokyo: • 1♂ (NHMUK), Katsushika-ku, Mizumoto Kôen Park, 8.V.2005, leg. Y. Komiya; Shikoku. Ehime: • 1♂ (HAPC), Kumakôgen-chô, Mt. Saragamine, 7.VI.2009, leg. H. Suenaga; • 1♂, 2♀ (HAPC), Matsuyama-shi, Mt. Takanawa-san, 12.V.2007, leg. S, Sejima; Kyushu. • 3♂, 1♀ (TARI), Mt. Hiko-san, 14.VIII.1941, leg. M. Chûjô; Fukuoka: • 2♂ (HAPC), Soeda-machi, Mt. Hiko-san, 8.VIII.2009, leg. S. Sejima; **Russian Far East.** Primorsky Krai: • 2♂ (NHMUK), Lazovski Zapovednik, 170 m E Vladivostok, Korpad, 28.V.-6.VI.2001, leg. M. Quest; • 1♂ (NHMUK), Odarkovskij, Zavod, 25.IV.1911, leg. A. Tsherskij; • 1♂ (NHMUK), Wladiwostok, leg. Herman Frieb.; **South Korea.** • 1♀ (TARI), Sulgen, 15.VII.1932, leg. D. Okamoto; **Taiwan.** Taipei: • 1♂, 1♀ (TARI), Kueitzukeng (貴仔坑), 4.XII.2006, leg. H.-T. Cheng; • 1♀ (TARI), same but with “leg. H. Lee”; • 1♂ (TARI), same locality, 9.IX.2007, leg. M.-H. Tsou; • 1♀ (TARI), same but with “18.XI.2007”; • 2♀ (TARI), Tienmu (天母), 8.XII.2006, leg. S.-F. Yu.

#### Diagnosis.

**Figure 3.**
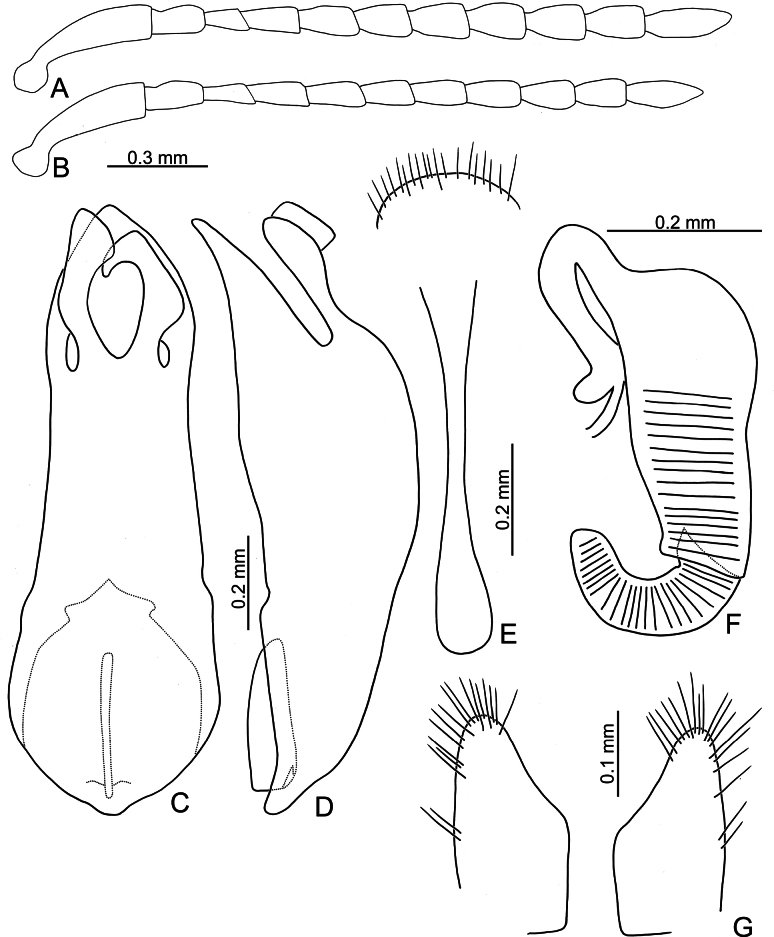
*Argopistesbiplagiatus* Motschulsky **A** antenna, male **B** antenna, female **C** aedeagus, dorsal view **D** aedeagus, lateral view **E** abdominal ventrite VIII, female **F** spermatheca **G** gonocoxae.

**Figure 4.**
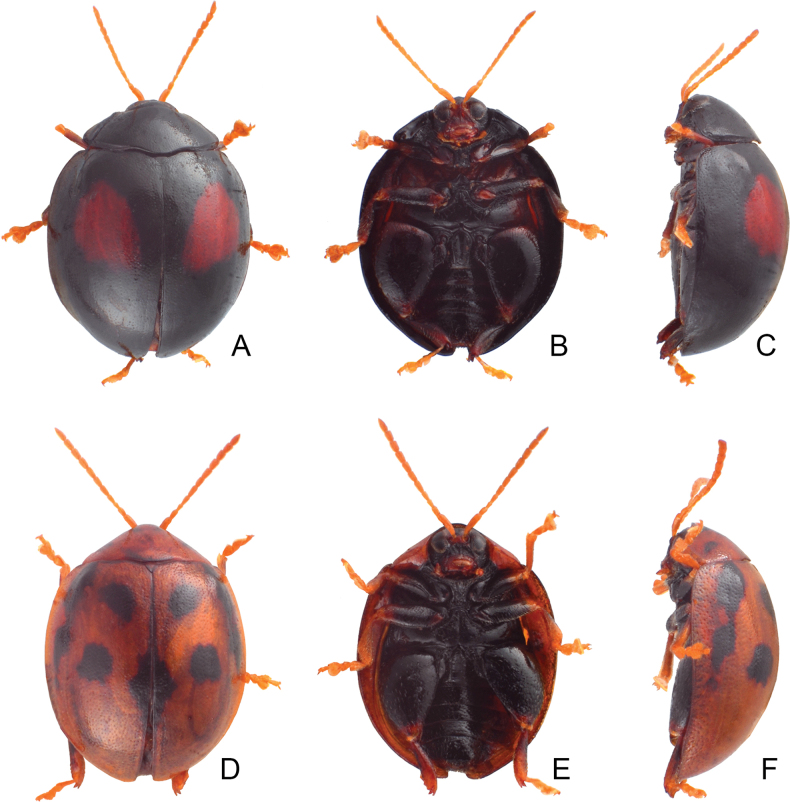
Habitus of *Argopistesbiplagiatus* Motschulsky **A** typical color form, female, dorsal view **B** ditto, ventral view **C** ditto, lateral view **D** yellowish brown color form, female, dorsal view **E** ditto, ventral view **F** ditto, lateral view.

**Figure 5.**
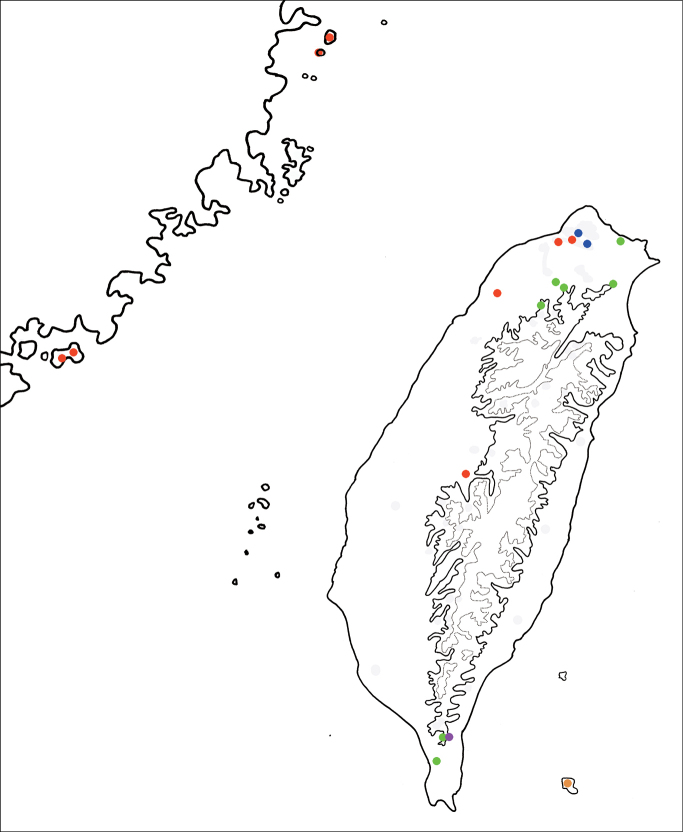
Distribution map of *Argopistes* species in Taiwan, solid line: 1000 m, broken line: 2000 m. Red dots *A.rufus* Chen; blue dots *A.biplagiatus* Motschulsky; green dots *A.tsoui* sp. nov.; orange dots *A.yuae* sp. nov.; purple dot *A.jungchani* sp. nov.

**Figure 6.**
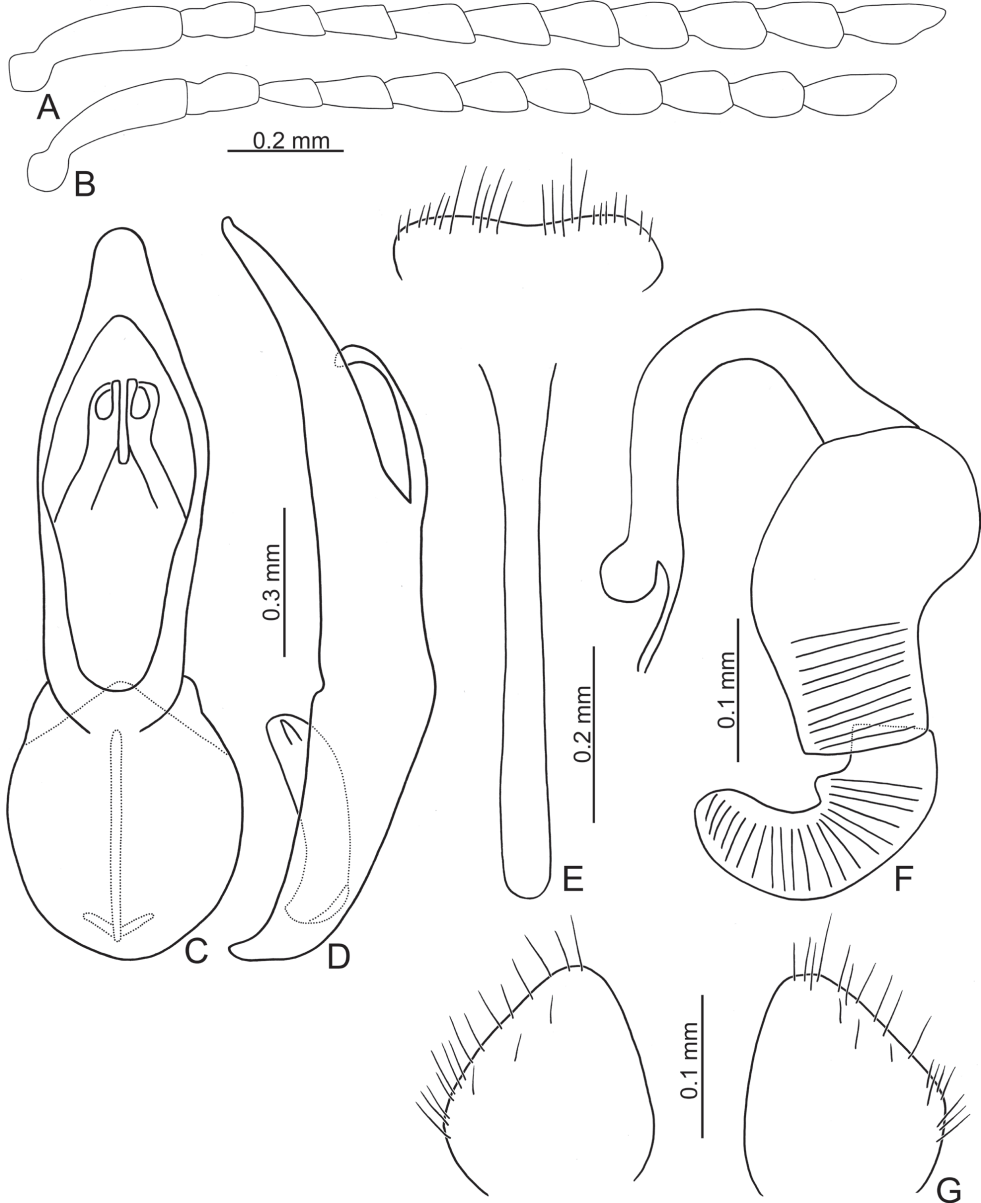
*Argopistesrufus* Chen **A** antenna, male **B** antenna, female **C** aedeagus, dorsal view **D** aedeagus, lateral view **E** abdominal ventrite VIII, female **F** spermatheca **G** gonocoxae.

Adults of *Argopistesbiplagiatus* are similar to those of *A.rufus* with similar color pattern but differing from *A.rufus* possessing line of punctures that are less coarse than those between the lines, sometimes confused (lines of punctures much coarser than those between lines in *A.rufus*) and a wider interspace between eyes. Genitalic characters are more diagnostic for both species. Those of *A.biplagiatus* possess pointed apices (Fig. 3C) and are wider in lateral view (Fig. 3D) (widely rounded apex (Fig. 5C) and narrow aedeagus in lateral view (Fig. 6D) in *A.rufus*); females have narrow, parallel-sided bases of gonocoxae (Fig. 3G) (medially widened gonocoxae (Fig. 5G) in *A.rufus*), and ventrite VIII evenly rounded and with dense setae on apical margin (Fig. 4E) (medially depressed and without setae on median area of apical margin of abdominal ventrite VIII (Fig. 5E) in *A.rufus*).

In addition, adults of *A.biplagiatus* in Taiwan are larger (4.7–4.9 mm) than those of *A.rufus* (3.8–4.3 mm). Moreover, distinct color patterns occur in both species respectively (black elytra with reddish brown at middle in *A.biplagiatus*; yellowish brown elytra with distinct arrangement of black spots in *A.rufus*).

#### Redescription.

Length 4.4–4.9 mm, width 3.5–3.8 mm. Color variable (see below). Pronotum broad, convex, lateral margin narrowly explanate; 2.0–2.2 × wider than long, disc with dense coarse punctures; lateral margin rounded, anterior margin strongly concave, posterior margin moderately convex. Intercoxal prosternal process flattened and with coarse punctures, delimited by narrow ridge on apical and lateral margins, truncate or slightly rounded at apex. Elytra broadly oval, 1.1 × longer than wide, disc with dense, confused, coarse punctures. Abdominal ventrite I with intercoxal area 2.0 × as long as wide, widest at basal 1/5, disc glabrous, rounded by reversed U-shaped ridge, provided with a row of coarse punctures inside subparallel lateral ridges.

**Male.** Antenna filiform (Fig. 3A), antennomere I much longer than others, approximate ratios of length of antennomeres I–XI 1.0: 0.3: 0.2: 0.4: 0.4: 0.3: 0.4: 0.4: 0.4: 0.4: 0.6; approximate ratios of length to width of antennomeres I–XI 4.4: 1.9: 1.7: 2.4: 2.0: 1.6: 1.6: 1.8: 1.7: 1.7: 2.9. Aedeagus (Fig. 3C, D) apically and strongly narrowed from apical 1/5, slightly narrowed from apical 2/10–3/10, then slightly and basally widened towards basal 1/6, apex pointed; anterior opening very small, from apex to apical 3/10; tectum composed of one pair of sclerotized processes with apices twisted; extremely wide and straight in lateral view; paired processes straight in lateral view; endophallic sclerite laterally flattened, with small process near apex, and with basal processes membranous.

**Female.** Antenna (Fig. 3B) similar to males, ratios of length of antennomeres I–XI 1.0: 0.3: 0.2: 0.3: 0.3: 0.3: 0.3: 0.3: 0.3: 0.3: 0.6; ratios of length to width of antennomeres I–XI 4.5: 2.0: 1.7: 2.0: 1.9: 1.7: 1.5: 1.6: 1.3: 1.5: 2.4. Ventrite VIII (Fig. 3E) membranous, only apical margin sclerotized, T-shaped, with dense long setae along apical margin, apical margin widely rounded, spiculum long. Spermathecal receptaculum (Fig. 3F) much longer than pump, moderately swollen, curved in lateral view; pump slightly emarginated at inner side of base; spermathecal duct with long basal part, ramus rounded. Gonocoxae (Fig. 3G) wide and separated, base membranous, each gonocoxa longitudinal and asymmetric, apically narrowed from middle, with dense long setae along apical areas.

#### Color variation.

In Japan, two distinct color patters of adults, typical color form (Fig. 4A–C): general color black, each elytron with one large red spot, lateral margin sometimes yellowish brown, legs dark brown but tarsi yellowish brown, head entirely black, or with one yellowish brown spot on vertex, or entirely yellowish brown except above eyes, abdominal ventrites yellowish brown but medially black; yellowish brown color form: general color yellowish brown (Fig. 4D–F; undecimmaculata form), pronotum with one pair of small lateral black spots, elytra with 11 black spots, two pairs arranged into transverse lines near base and middle, one transverse pair near suture at middle, others longitudinal, one additional transverse pair near apex, one spot along suture from basal 1/3 to apical 1/3, medially widened, head yellowish brown but black below eyes except mouthparts, thoracic and abdominal ventrites black but abdominal ventrites laterally yellowish brown, legs black but tarsi, pro- and mesotibiae yellowish brown.

At the type locality (Russian Far East and northeastern China), some individuals represent the typical form (Fig. 2B) but with yellowish margins of pronotum and elytra, some with enlarged red spots on the elytra connected with each other, some with entirely yellowish-brown bodies (Fig. 2C).

In Taiwan, some specimens represent the typical form, but some have enlarged red spots on elytra that extend into the basal margin and connect with each other, and have reddish brown thoracic and abdominal ventrites.

#### Host plants.

Inoue (1990a) recorded the following species as host plants: Osmanthus×fortunei, *O.heterophyllus*, *O.fragrans* (桂花), O.fragransvar.aurantiacus Makino, *Ligustrumjaponicum* (日本女真), *L.ovalifolium* Hassk., *L.licidum* W. T. Aiton, *Syringavulgaris* L., and *S.reticulata* (Blume) H. Hara. Chûjô and Kimoto (1961) recorded one additional host, FraxinusmandshuricaRupr.var.japonica Maxim. Lee and Cho (2006) recorded *Ligustrumobtusifolium* Siebold & Zucc for Korean populations.

#### Biology.

Various aspects of biology of *A.biplagiatus* were studied in Japan, including feeding habits, seasonal development, habitat selection, host plant preference, and adult diapause (Inoue 1990a, b, 1991b, 1992, 1993, 1994). Generally, the species has a univoltine life cycle. Eggs and/or larvae of this species are observed in the spring. Mature larvae fall from the host trees rather than crawling (Inoue 2014).

#### Remarks.

Syntypes of *A.biplagiatus* Motschulsky display great color variation. Several names (*A.flavitarsis*, *A.limbatus*, and *A.suturalis*) have been proposed for different color patterns.

#### Distribution.

China, Japan (Hokkaido, Honshu, Shikoku, Kyushu), Russian Far East, South Korea, and new to Taiwan (Fig. 5).

### ﻿ Argopistes rufus

Taxon classificationAnimaliaColeopteraChrysomelidae

Chen, 1934 stat. nov.

FB6FB5E2-5732-5404-9241-4BF16355B8FA

**Figure 7.**
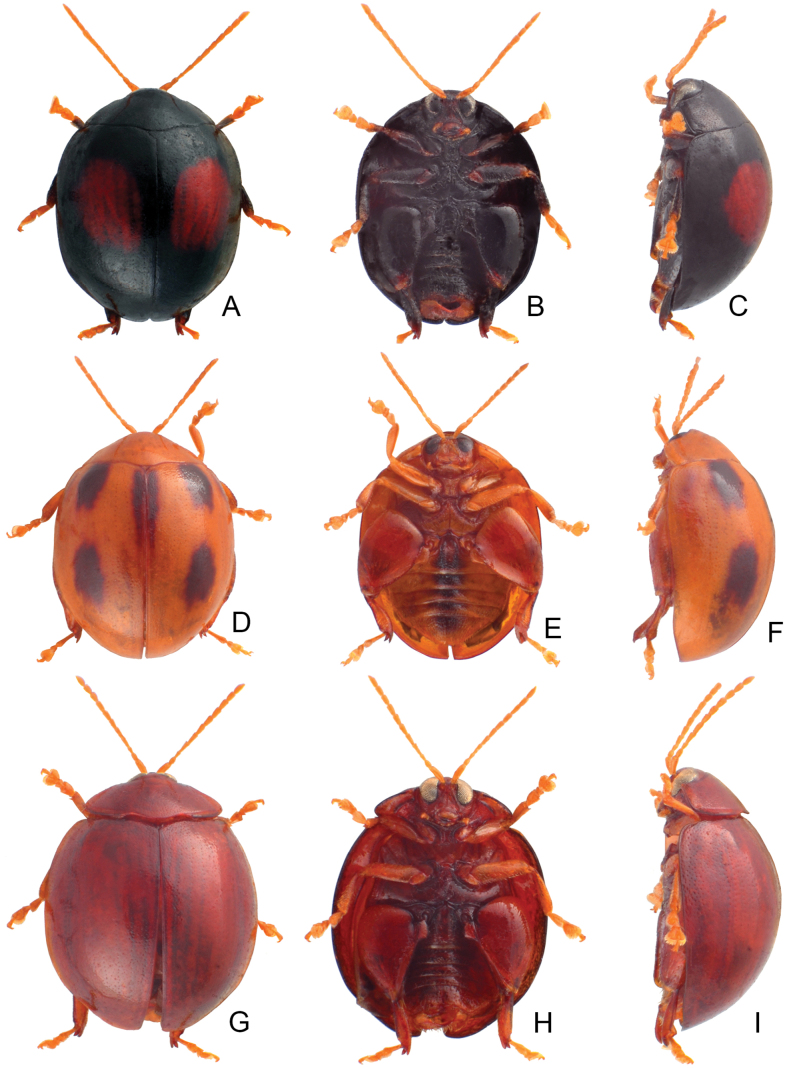
Habitus of *Argopistesrufus* Chen **A** typical color form, male, dorsal view **B** ditto, ventral view **C** ditto, lateral view **D** yellowish brown color form, female, dorsal view **E** ditto, ventral view **F** ditto, lateral view **G** reddish brown color form, male, dorsal view **H** ditto, ventral view **I** ditto, lateral view.

Figs 1 , 2G, H , 6 , 7

Argopistes coccinelloides Baly, 1874 (nec Suffrian, 1868): 202 (Japan); Chûjô 1935a: 87 (Japan: Okinawa); Chûjô 1935b: 211 (catalogue).
Argopistes biplagiatus : Schönfeldt 1890: 175 (Japan: Loochoo); Chûjô 1936: 110 (Taiwan), misidentification (Gressitt and Kimoto 1963).
Argopistes biplagiatus var. rufus Chen, 1934a: 72 (China).
Argopistes coccinelliformis Csiki, 1940: 524 (new replacement name for A.coccinelloides Baly, 1874); Chûjô and Kimoto 1961: 174 (catalogue); Gressitt and Kimoto 1963: 812 (South China); Kimoto 1965: 436 (redescription); Takizwa, 2012: 38 (faunistics).
Argopistes ryukyuensis Shigetoh & Suenaga, 2022: 4 (Japan: Okinawa). syn. nov.

#### Type material examined.

*Argopistescoccinelloides*. ***Holotype*** • (sex undetermined, NHMUK) (Fig. 2G, H): “Argopistes / coccinelloides / Baly / Japan [h, b] // Type / H.T. [p, circle label with red border] // Baly Coll. [h, w] // BMMH(E) / #1024843 [p, w]”.

Argopistesbiplagiatusvar.rufus. ***Lectotype*** • ♀ (here designated, NHMUK): “China [p] // Bowring / 63•37* [p] // Argopistescoccinelliformis / Csiki, 1940 / det C.-F. Lee, 2023 [p] ♀ [h, w] // NHMUK 015998267 [with OR Code, p, w]”. ***Paralectoypes*** • 3♀ (NHMUK), same as lectotype but with “0155998268–0155998270”.

*Argopistesryukyuensis*. ***Paratypes*.** Japan: Kitadaitô-jima Island (北大東島): • 1♂, 3♀ (HAPC), Kitadaitô-jima, 21.IV.2018, leg. H. Kawase; Okinawa-jima Island: • 1♂ (HAPC), Tomigusuku-shi, Tomigusuku, 10.V.2020, leg. H. Shigetoh; 1 ♂, 1♀ (HAPC), same but with “11.III.2021”; Ou-jima Island: • 1♀ (HIPC), Nanjo-shi, Tamashiro-ou, 6.V.2019, leg. H. Shigetoh; • 3♂, 1♀ (1♂: HAPC; 2♂, 1♀: HIPC), same but with “2.III.2021”; Tonaki-jima Island: • 1♂, 1♀ (HIPC), Tonaki-son, Uaki, 1.IX.2018, leg. H. Shigetoh; Tsuken-jima Island: • 1♀ (HAPC), Uruma-shi, Katsurentsuken, 14-16.VII.2020, leg. H. Shigetoh; Yonaguni-jima Island: • 1♂, 1♀ (HAPC), Kita-Bokujô, 28.III.2001, leg. S. Tsuyuki.

#### Additional material examined.

**China.** Guandong: • 7♂, 6♀ (TARI), Yangtaishan (阳台山), 23.IV.2022, leg. Y.-Y. Ruan; • 13♂, 11♀ (TARI), Wutongshan (梧桐山), 5.IV.2023, leg. Y.-Y. Ruan; Hong Kong: • 2♂, 1♀ (NHMUK), 56 / 157, 894 / 7/8/63; • 1♂ (NHMUK), Walker Coll., 93—58; • 1♂ (NHMUK), Tailung National Park, 12.III.1963, leg. P. Y. So; **Japan.** Honshu. Gumma: • 1♀ (NHMUK), Maebashi-shi, Iwakami-chô, 18.IV.2003, leg. Y. Komiya; Okayama: • 1♀ (HAPC), Mimasaka-shi, Yono, 10.IV.2016, leg. H. Suenaga; • 1♂ (HAPC), Okayama-shi, Kita-ku, Kibi service area, 1.VII.2012, leg. O. Yamaji; • 2♂, 2♀ (HAPC), Tsuyama-shi, Yamakita, 3.IV.2014, leg. H. Suenaga; Tokyo: • 3♂, 3♀ (HIPC), Hachiôji-shi, Kinugaoka, 18.VI.2016, leg. H. Shigetoh; Hachijô-jima Island: • 2♂, 2♀ (SEHU), Okagô, 3.VIII.1963, leg. Y. Kamiya; Ogasawara Haha-jima Island: • 1♂ (SEHU), Funamidai, 24.VI.1987, leg. H. Akiyama; Kyushu. Fukuoka: • 1♀ (HAPC), Fukuoka-shi, Higashi-ku, Hakozaki Kyushu Univ., 7.VI.2008, leg. Y. Matsumura; • 1♀ (HAPC), Fukuoka-shi, Hakozaki, 16.VIII.2011, leg. H. Suenaga; Kagoshima: Koshiki-jima Island: • 1♀ (SEHU), Teuchi, 16.V.1965, leg. Y. Komiya; the Ryukyus. Okinawa: Kita-daitô-jima Island: • 2♂, 2♀ (HIPC), Kita-daitô-jima, 21.IV.2018, leg. H. Kawase; **Taiwan.** Hsinchu: • 3♂, 6♀ (TARI), Hsinchu (新竹市), 23.IV.2021, leg. C.-Y. Tsai; Kinmen: Kinmen Island (金門島): • 1♀ (TARI), Botanic Park (植物園), 12.VII.2023, leg. C.-F. Lee; • 3♂, 14♀ (TARI), Jinsha (金沙), 12.IV.2023, leg. C.-F. Lee; Matsu Islands: • 10♂, 11♀ (TARI), Beigan Island (北竿島), 12.IV.2024, leg. C.-F. Lee; • 6♂, 10♀ (TARI), Nangan Island (南竿島), 12.IV.2024, leg. C.-F. Lee; Nantou: • 4♂, 5♀ (TARI), Chichi (集集), 26.V.2023, leg. T.-W. Hsu; • 2♂ (TARI), Mingchien (名間), 14.VII.2022, leg. Y.-J. Tung; Taipei: • 1♂, 1♀ (TARI), Kuantu (關渡), 8.IV.2020, leg. M.-H. Tsou; • 4♂, 1♀ (TARI), same locality, 18.X.2010, leg. S.-F. Yu; • 1♀ (TARI), same but with “20.II.2011”; • 5♂, 6♀ (TARI), Kuanyinshan (觀音山), 21.III.2016, leg. H.-T. Cheng; • 2♂ (TARI), same locality, 20.V.2011, leg. H. Lee.

#### Diagnosis.

Adults of *A.rufus* look similar to those of *A.biplagiatus* with a similar color pattern, but differ from *A.biplagiatus* in having lines of punctures much coarser than those between the lines (lined punctures slightly coarser than those between lines, sometime confused in *A.biplagiatus*) and a narrower interspace between eyes. Genitalic characters are diagnostic for both species. Those of *A.rufus* possess widely rounded apices (Fig. 6C) and the aedeagus is narrow in lateral view (Fig. 6D) (pointed apex (Fig. 3C) and wider aedeagus in lateral view (Fig. 3D) in *A.biplagiatus*); females have medially widened gonocoxae (Fig. 6G) (narrow and parallel-sided base of gonocoxae (Fig. 3G) in *A.biplagiatus*), and abdominal ventrite VIII medially depressed and without setae on median area (Fig. 6E) (evenly rounded and with dense setae on apical margin of abdominal ventrite VIII (Fig. 3E) in *A.biplagiatus*).

In addition, adults of *A.rufus* in Taiwan are smaller (3.8–4.3 mm) than those of *A.biplagiatus* (4.7–4.9 mm). Moreover, distinct color patterns occur to both species respectively (yellowish brown elytra with distinct arrangement of black spots in *A.rufus*; black elytra with reddish brown central area in *A.biplagiatus*).

#### Redescription.

Length 3.6–4.3 mm, width 2.9–3.4 mm. Color variable (see below). Pronotum broad, convex, lateral margin narrowly explanate; 2.1–2.2 × wider than long, disc with dense fine punctures; lateral margin rounded, anterior margin strongly concave, posterior margin moderately convex. Elytra broadly oval, 1.1 × longer than wide, disc with coarse punctures arranged into longitudinal striae and with dense fine punctures between striae.

**Male.** Antenna filiform (Fig. 6A), antennomere I much longer than others, approximate ratios of length of antennomeres I–XI 1.0: 0.4: 0.3: 0.4: 0.4: 0.4: 0.4: 0.4: 0.4: 0.4: 0.5; approximate ratios of length to width of antennomeres I–XI 4.3: 2.0: 2.0: 2.0: 2.0: 1.8: 1.5: 1.6: 1.7: 1.7: 2.7. Aedeagus (Fig. 6C, D) apically and strongly narrowed from apical 1/3, apex truncate; anterior opening large, ~ 0.52 as long as aedeagus, from apical 1/8–3/5; tectum composed of one pair of sclerotized processes with bifurcate apices, outer apex hooked, small, ~ 0.36 as long as anterior opening; narrow and slightly curved in lateral view; paired processes apically curved in lateral view; endophallic sclerite laterally flattened, with small process near apex, and with basal processes.

**Female.** Antenna (Fig. 6B) similar to males, ratios of length of antennomeres I–XI 1.0: 0.4: 0.3: 0.4: 0.4: 0.3: 0.3: 0.4: 0.4: 0.4: 0.5; ratios of length to width of antennomeres III–XI 4.4: 1.9: 2.0: 2.2: 1.8: 1.6: 1.4: 1.7: 1.6: 1.6: 2.3. Ventrite VIII (Fig. 5E) weakly sclerotized, T-shaped, with several setae along apical margin, apical margin medially depressed, spiculum long. Spermathecal receptaculum (Fig. 6F) longer than pump, moderately swollen, curved in lateral view; pump emarginate at inner side of base; spermathecal duct with long basal part, ramus rounded. Gonocoxae (Fig. 6G) wide and separated, base membranous, each gonocoxa asymmetric, apically narrowed from basal 1/3, with sparse long setae along apical areas.

#### Color variation.

In Japanese populations, antennae yellowish brown; pronotum and elytra black, each elytron with one large red spot, sometimes widened and spots connected to each other, red spot reduced in some individuals; mesoventrite and abdominal ventrites reddish brown but medially black; femora blackish brown, tibiae dark brown, tarsi yellowish brown; few individuals have entirely reddish-brown bodies. In the Ryukyus, adults usually have larger red spots on the elytra and reddish-brown elytral margins (described as *A.ryukyuensis* Shigetoh & Suenaga, 2022).

On Taiwan Island, adults separate into two color forms. Typical form (Fig. 7A–C): black elytron with one large red spot, same as Japanese populations; yellowish brown color form (Fig. 7D–F): elytra with wide black stripe along suture, starting from base, apically narrowed and abbreviated at basal 1/3, with two pairs of black spots halfway between suture and lateral margin, anterior pair at base, posterior pair at apical 1/3, one wide black stripe along lateral margin, starting from base, apically narrowed, abbreviated at basal 1/3 or 1/4; abdominal ventrites medially darker. This color form is also found in Nangan Island.

In China and Kinmen Island, almost all adults belong to the typical form. A few specimens have entirely reddish-brown bodies (Fig. 7G–I). Three specimens collected from Hong Kong also have reddish bodies.

#### Host plants.

Inoue (2014) recorded Osmanthus×fortunei, *O.heterophyllus*, *O.fragrans* (桂花), O.fragransvar.aurantiacus, *O.insularis* Koidz, *Ligustrumjaponicum* (日本女真), *L.licidum*, *L.ovalifolium*, *L.obtusifolium*, *Syringavulgaris*, *S.reticulata*, *Jasminumnudiflorum* Lindl., and *Oleaeuropaea* L. as host plants in Japan. In Taiwan, larvae mine the leaves of the following plants: *Chionanthusretusus* (in Taiwan Island, Nangan, and Beigan islands), *Ligustrumjaponicum* (in Beigan island), and *Osmanthusfragrans* (in Kinmen Island).

#### Biology.

Various aspects of the biology of *A.rufus* were studied in Japan, including feeding habits, habitat selection, seasonal development, and developmental biology on various host trees, developmental success of larvae on two different host trees, seasonal trends of feeding and oviposition activities of adults, effects of food condition on oviposition, overwintering and oviposition ability of adults that emerge late in the season, effects of photoperiod and temperature on induction of reproductive diapause in newly emergence adults, and occurrence on olive trees (Inoue and Shinkaji 1989a–c, 1990; Inoue 1990b, 1991a, 1998, 2001, 2014).

The seasonal development of this species was studied in the field in southern Kantô, Central Japan (Inoue 1996). Overwintered adults appeared on host trees beginning mid-March, with a peak in mid-April to early May. Females began to deposit eggs from mid- to late April. The eggs were laid singly, embedded in young leaves, and coated with excrement. Leaf-mining larvae only developed in new leaves. Larvae underwent three larval instars and mature larvae crawled down to pupate in the upper layers of soil. Adults eclosed in mid-June, with a peak in later June-early July. They mainly fed on mature leaves. Adults passed the winter near the ground, mainly under fallen leaves. The egg, larval, prepupal, and pupal period took ~ 10, 20–30, 10–15, and 10–15 days respectively during spring to early summer. In Taiwan, larvae and adults can be found during April.

#### Remarks.

Argopistesbiplagiatusvar.rufus was described by Chen (1934a) based on four reddish brown individuals (Fig. 7G–I) deposited in the NMHUK. We found the determination label: “Argopistes / biplagiatus / var. rufa”, handwritten by Chen pinned with one typical form (Fig. 7A–C). Four adjacent females fit the original description (reddish brown body form) and bore two labels “China / Bowring” although no determination labels were found. Thus, those specimens were designated as lectotypes and paralectotypes. Bezděk and Konstantinov (2024) placed this name as a junior synonym of *A.coccinelliformis* Csiki, 1940. Actually, it is a distinct species and attributed to the oldest available name. Thus, the valid name is *Argopistesrufus* Chen, 1934, stat. nov.

Adults of *A.rufus* and *A.ryukyuensis* are not separable when Taiwanese and Chinese specimens are included. Aedeagi of both areas are intermediate between *A.rufus* and *A.ryukyuensis*. Moreover, one distinct color pattern (yellowish-brown elytra with black spots) occurs in Taiwanese populations. Thus, color patterns may not be considered as diagnostic characters. Other diagnostic characters provided by Shigetoh and Suenaga (2022) are not diagnostic for species delimitation. Thus *A.ryukyuensis* Shigetoh & Suenaga, 2022 is regarded as junior synonym of *A.rufus* Chen, 1934.

#### Distribution.

China, Japan (Honshu, the Izu Isls., Ogasawara Isls., Shikoku, Kyushu, Okinoshima Is., Kashiwa-jima Is., the Koshiki-jima Isls., Yakushima Is., the Ryukyu Isls.), Taiwan including Kinmen Island and Matsu Islands (Beigan and Nangan Islands) (Fig. 5).

### ﻿ Argopistes tsekooni

Taxon classificationAnimaliaColeopteraChrysomelidae

Chen, 1934

CF894C90-4375-52B8-B9F4-123697977F97

Figs 8A–C , 9

Argopistes tsekooni Chen, 1934b: 316 (China: Shanghai, Hangchow); Csiki 1940: 525 (catalogue); Chûjô and Kimoto 1961: 174 (China, Japan); Gressitt and Kimoto 1963: 813 (China: Jiangsu); Kimoto 1965: 437 (redescription); Lee and An 2001: 183 (South Korea); Lee and Cho 2006: 91 (host plant); Takizwa, 2012: 38 (faunistics); Cho and An 2020: 15 (North Korea); Won et al. 2023: 9 (South Korea: Ulleungdo).
Argopistes biplagiatus : Baly 1874: 202 (misidentification).

#### Type material examined.

One ***syntype*** • (sex undetermined, IZAS): “浙江 (= Zhejiang): 杭州 (= Hangchow) / 1934. [h] / 中国科學院 (= Chinese Academy of Sciences) [h, p] // 害水蜡樹 (attacking *Ligustrumobtusifolium*) [h, w] // Argopistes / tsekooni / Chen [h, w]”. Although this specimen does not bear any type label, it should be regarded as type specimen since it fit the original description.

#### Additional material examined.

**Japan.** • 1♀ (NHMUK): “Argopistes / biplagiatus / Motsch / Japan [h, w] // Baly Coll. [p, w]”; Honshu. Shizuoka: • 1♂ (SEHU), Tagata-gun, Tohi, 4.V.1985, leg. Y. Komiya; Tokyo: • 1♂ (HAPC), Komae-shi, Komai-machi, 10.VI.2021, leg. R. Seki; Yamaguchi: • 1♂ (NHMUK); Kyushu. Fukuoka: • 1♀ (HAPC), Fukuoka-shi, Higashi-ku, Shimobaru (alt. 100–360 m), 27.V.2009, leg. S. Sejima; • 1♀ (NHMUK), Mt. Mikazuki, 2.V.1954, leg. K. Morimoto; Nagasaki: • 1♂, 2♀ (SEHU), Sasebo-shi, Mt. Yahirodake, 14.IV.1981, leg. J. Okuma; • 1♂ (SEHU), same locality but with “21.IV.1981”; Oita: • 2♂, 3♀ (HAPC), Hita-shi, Miwa, Chikura, 11.IV.2016, leg. S. Sasaki.

#### Diagnosis.

Adults of *A.tsekooni* are recognized easily by their small body sizes (< 3.5 mm; > 3.5 mm in others except *A.unicolor*), elongate ovate body shapes (elytra 1.2 × longer than wide; but 1.1 × longer than wide in others), and the combined red spots on elytra (usually separate red spots on the elytra in others); additionally, most genitalic characters are unique, such as the tube-like apex of the aedeagus (Fig. 9C); few setae on apical margin of abdominal ventrite VIII in females (Fig. 9E); and transverse gonocoxae with dense, long setae on the widely rounded apical margin (Fig. 9G).

#### Redescription.

**Figure 8.**
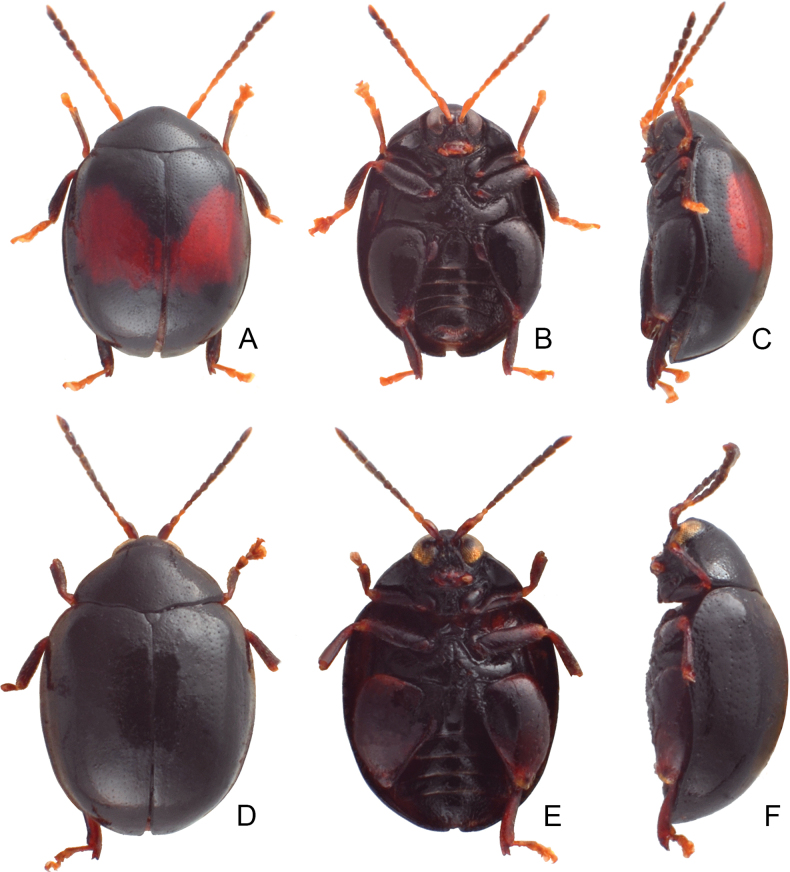
Habitus of *Argopistes* species **A***A.tsekooni* Chen, male, dorsal view **B** ditto, ventral view **C** ditto, lateral view **D***A.unicolor* Jacoby, female, dorsal view **E** ditto, ventral view **F** ditto, lateral view.

Length 2.8–3.2 mm, width 2.1–2.4 mm. Color (Fig. 8A–C) blackish brown, elytron with one transverse orange area at basal 1/3, and narrowed towards suture; tarsi and front tibiae yellow; antennae dark brown but seven basal antennomeres yellow. Pronotum broad, convex, lateral margin narrowly explanate; 2.0–2.1 × wider than long, disc with dense coarse punctures; lateral margin rounded, anterior margin strongly concave, posterior margin moderately convex. Elytra elongate oval, 1.2 × longer than wide, disc with confused, dense, coarse punctures.

**Figure 9.**
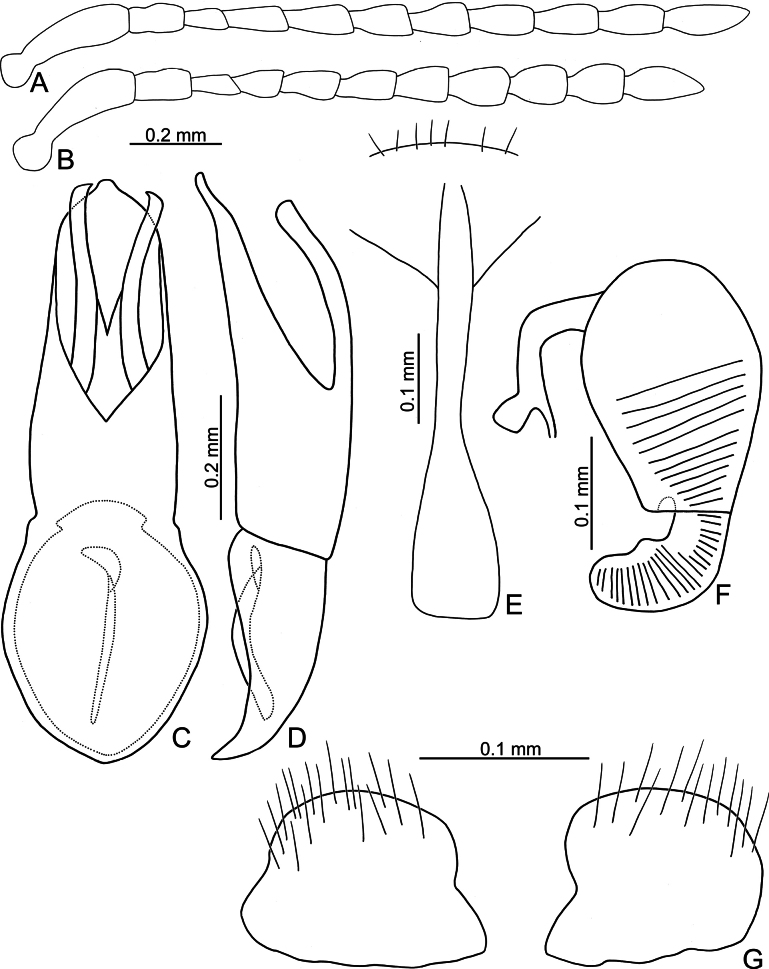
*Argopistestsekooni* Chen **A** antenna, male **B** antenna, female **C** aedeagus, dorsal view **D** aedeagus, lateral view **E** abdominal ventrite VIII, female **F** spermatheca **G** gonocoxae.

**Male.** Antenna filiform (Fig. 9A), antennomere I much longer than others, approximate ratios of length of antennomeres I–XI 1.0: 0.4: 0.3: 0.5: 0.5: 0.5: 0.5: 0.5: 0.5: 0.5: 0.7; approximate ratios of length to width of antennomeres I–XI 3.1: 1.8: 2.0: 2.5: 2.3: 1.9: 1.8: 2.1: 2.1: 2.2: 2.9. Aedeagus (Fig. 9C, D) gradually widened from basal 1/9–1/2, then gradually narrowed to basal 1/2, strongly widened posterior–basal 1/2; anterior opening large, ~ 0.39 as long as aedeagus, from apex to apical 2/5; tectum composed of one pair of sclerotized processes, long, ~ 0.85 as long as anterior opening, wide and slightly curved from basal 2/4 to apex in lateral view, recurved near apex; endophallic sclerite laterally flattened, with base twisted.

**Female.** Antenna (Fig. 9B) similar to males, but antennomeres VII–X wider, ratios of length of antennomeres I–XI 1.0: 0.4: 0.3: 0.4: 0.4: 0.4: 0.4: 0.4: 0.4: 0.4: 0.6; ratios of length to width of antennomeres I–XI 3.8: 1.8: 2.0: 1.8: 1.9: 1.9: 1.6: 1.5: 1.5: 1.5: 2.4. Ventrite VIII (Fig. 9E) weakly sclerotized, only part of apical margin well sclerotized, with several setae along apical margin, spiculum long and base wider. Spermathecal receptaculum (Fig. 9F) longer than pump, moderately swollen, curved in lateral view; pump emarginated at inner side of base; spermathecal duct with long basal part, ramus truncate. Gonocoxae (Fig. 9G) wide and separated, base membranous, each gonocoxa asymmetric, apically narrowed from near base, with sparse setae along apical areas, setae longer at apical 1/2.

#### Color variation.

One male has a black body and lacks transparent spots on elytra. Another male has an entire yellowish-brown body.

#### Host plants.

Oleaceae: *Ligustrumobtusifolium* (Chûjô & Kimoto, 1961); *Syringaoblata* Lindl., *L.japonicum*, *L.licidum*, and *L.sinense* (Zhang et al. 2008b).

#### Biology.

The biology and life history of *A.tsekooni* were studied under laboratory and outdoor conditions in Huangshan City of Anhui Province, China (Zhang et al. 2009). *Argopistestsekooni* overwintered as adults and had three overlapping generations in Anhui Province.

#### Distribution.

China, Japan (Honshu, Kyushu, the Goto Isls., Hirado-jima Is.Tsushima Is.), North Korea, South Korea.

### ﻿ Argopistes unicolor

Taxon classificationAnimaliaColeopteraChrysomelidae

Jacoby, 1885

4DC625D6-15FE-50CE-B9F7-AA0871B5A9FC

Figs 8D–F , 10

Argopistes unicolor Jacoby, 1885: 738 (Japan: Yuyama); Chûjô 1936: 109 (catalogue); Csiki 1940: 524 (catalogue); Chûjô and Kimoto 1961: 174 (catalogue); Kimoto 1965: 438 (redescription); Takizawa, 2012: 38 (faunistics).

#### Type material examined.

***Lectotype*** • ♂ (here designated, NHMUK): “(aedeagus glued on the transparent card) // Yuyama / 10.V.-14.V.81. [p, w] // Japan / G. Lewis. / 1910-320 [p, w] // Type / H.T. [p, w, circle label with red border] // Argopistes / unicolor. Jac. [h, b] // Argopistes unicolor JACOBY, / LECTOTYPUS 1885 / J. Král m. dit 1969! [h, w] // lecto- / typus [p, r]”. ***Paralectotype*.** • 1♂ (TARI): “Yuyama [h] / Japan [p] / 10.V.1881 [h] / Col. G. LEWIS [p, w] // Argopistes / unicolor Jac. [h] / Det. T. Shiraki [p, w] // Co / Type [p, w, circle label with yellow letters and border] // Argopistes / unicolor Jacoby [h] // DET. M. CHUJO [p, w] // 1527 [p, w]”.

#### Additional material examined.

**Japan.** Kyushu. Nagasaki: • 3♂, 3♀ (SEHU), Shimbara-shi, Senbuki, 8.V.1984, leg. S. Imsaka; • 2♂, 2♀ (HAPC), same but with “Senfujiki”, collected on *Ligustrumjaponicum*.

#### Diagnosis.

**Figure 10.**
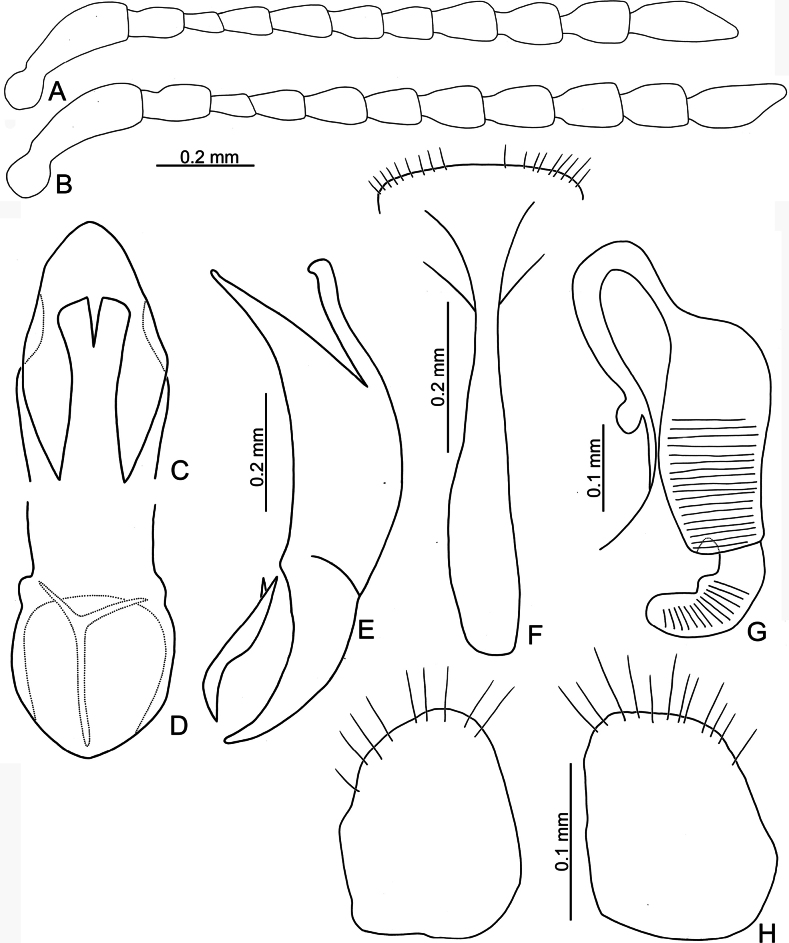
*Argopistesunicolor* Jacoby **A** antenna, male **B** antenna, female **C** apex of aedeagus, front view **D** base of aedeagus, dorsal view **E** aedeagus, lateral view **F** abdominal ventrite VIII, female **G** spermatheca **H** gonocoxae.

Adults of *A.unicolor* are recognized easily by their small body sizes (< 3.5 mm; > 3.5 mm in others except *A.tsekooni*), black antenna with three basal antennomeres paler (entirely yellowish-brown antennae in others except *A.tsekooni* with five dark apical antennomeres), and the entirely black elytra. Additionally, most genitalic characters are unique, including strongly curved aedeagus in lateral view (Fig. 10E), and anterior opening from apex to middle (Fig. 10C); straight apical margin of abdominal ventrite VIII in females (Fig. 10F) with setae reduced at medial area (other species with straight margin of abdominal ventrite VIII in female always with setae on median area); and longitudinally square gonocoxae (Fig. 10H).

#### Redescription.

Length 3.2–3.4 mm, width 2.3–2.5 mm. Color (Fig. 8D–F) black; legs and mouthparts dark brown; antenna black but three basal antennomeres dark brown; abdominal ventrites yellowish brown but medially darkened. Pronotum broad, convex, lateral margins narrowly explanate; 1.9–2.0 × wider than long, disc with dense coarse punctures; lateral margin rounded, anterior margin strongly concave, posterior margin moderately convex. Elytra broadly oval, 1.1 × longer than wide, disc with coarse punctures arranged into longitudinal striae, and with fine punctures between striae.

**Male.** Antenna filiform (Fig. 10A), antennomere I much longer than others, approximate ratios of length of antennomeres I–XI 1.0: 0.4: 0.3: 0.4: 0.4: 0.4: 0.4: 0.4: 0.4: 0.5: 0.7; approximate ratio of length to width of antennomeres I–XI 3.8: 1.8: 1.8: 2.0: 1.8: 1.6: 1.6: 1.7: 1.5: 1.6: 2.7. Aedeagus (Fig. 10C–E) widest at apical 1/4, slightly narrowed at middle, apically narrowed from apical 1/4, apex broadly rounded; anterior opening large, ~ 0.45 as long as aedeagus, from apex to middle; tectum composed of one pair of sclerotized processes, long, ~ 0.78 as long as anterior opening, paired processes with apices recurved in lateral view; endophallic sclerite laterally flattened, with one pair of long apical processes.

**Female.** Antenna (Fig. 10B) similar to males, ratios of length of antennomeres I–XI 1.0: 0.4: 0.3: 0.3: 0.4: 0.3: 0.4: 0.4: 0.4: 0.4: 0.6; ratios of length to width of antennomeres III–XI 3.4: 1.9: 1.9: 1.9: 1.7: 1.5: 1.6: 1.7: 1.5: 1.6: 2.4. Ventrite VIII (Fig. 10F) weakly sclerotized, T-shaped, with several pairs of setae along apical margin, spiculum long. Spermathecal receptaculum (Fig. 10G) longer and wider than pump, moderately swollen; pump emarginate at inner side of base; spermathecal duct with long basal part, ramus rounded. Gonocoxae (Fig. 10H) wide and separated, base membranous, each gonocoxa subquadrate, with sparse setae along apical areas.

#### Color variation.

One male has a black body and lacks transparent spots on the elytra. Another male has an entire yellowish-brown body.

#### Host plants.

Oleaceae: *Osmanthusheterophyllus* (= *Oleailicifolia* Hassk.) (Chûjô and Kimoto 1961), *Ligustrumjaponicum* (based on collecting data).

#### Biology.

Unknown.

#### Distribution.

Japan (Honshu, Kyushu, Hirado-jima Is.).

### ﻿ Argopistes jungchani sp. nov.

Taxon classificationAnimaliaColeopteraChrysomelidae

57009083-7C86-5358-A6D5-8687DA1C053F

https://zoobank.org/313C2711-2603-4D4D-A690-8E060F85480C

Figs 11A, B , 12

#### Type material examined.

***Holotype*** • ♂ (TARI). Taiwan. Pingtung: Jinshuiying (浸水營), 14.IV.2021, leg. J.-C. Chen. ***Paratype*** • 1♀ (TARI), same but with “27.VI.2012”.

#### Diagnosis.

Adults of this new species are easily recognized by their color pattern: black bodies with yellowish-brown lateral margins of pronotum and elytra; also, genitalic characters are diagnostic: tube-like apex of aedeagus similar to that of *A.tsekooni* but parallel-sided from near apex to middle (Fig. 12C) (apically narrowed from near apex to middle (Fig. 9C) in *A.tsekooni*), paired elongate tectum small, 0.76 as long as anterior opening (Fig. 12C) (paired elongate tectum long, 0.85 as long as anterior opening (Fig. 9C) in *A.tsekooni*), and anterior opening from apical 1/13–2/5 (Fig. 12C) (anterior opening from apex to apical 2/5 (Fig. 9C) in *A.tsekooni*); only two pair of long setae on apical margin of abdominal ventrite VIII (Fig. 12E) in females (more than two pair of setae on apical margin of abdominal ventrite VIII in females of other species), and cylindrical gonocoxae (Fig. 12G).

**Figure 11.**
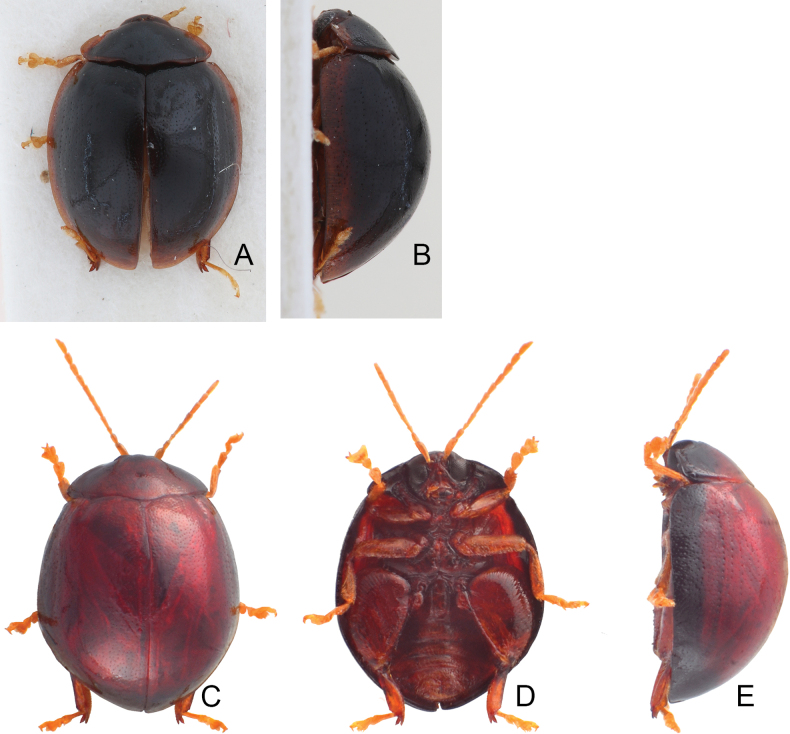
Habitus of *Argopistes* species **A***A.jungchani* sp. nov., female, dorsal view **B** ditto, lateral view **C***A.tsoui* sp. nov., male, dorsal view **D** ditto, ventral view **E** ditto, lateral view.

**Figure 12.**
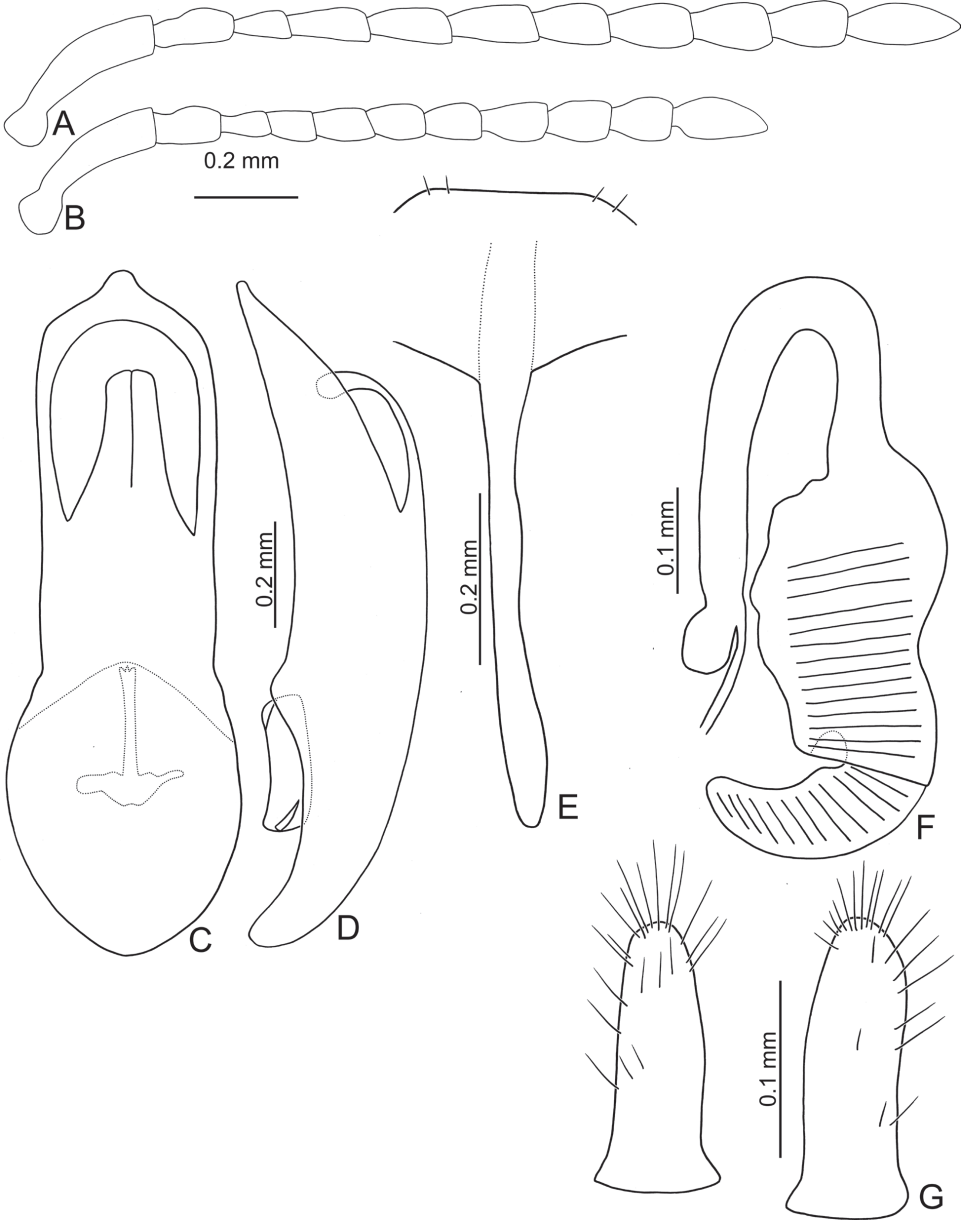
*Argopistesjungchani* sp. nov. **A** antenna, male **B** antenna, female **C** aedeagus, dorsal view **D** aedeagus, lateral view **E** abdominal ventrite VIII, female **F** spermatheca **G** gonocoxae.

#### Description.

Length 3.5–3.6 mm, width 2.7–2.9 mm. Color (Fig. 11A, B) blackish brown, sides of pronotum and elytra paled, tarsi, front femur and tibiae, and antennae yellowish brown. Pronotum broad, convex, lateral margin narrowly explanate; 2.3 × wider than long, disc with dense coarse punctures; lateral margin rounded, anterior margin strongly concave, posterior margin moderately convex. Elytra broadly oval, 1.1 × longer than wide, disc with fine punctures arranged into longitudinal lines, confused, dense, fine punctures present between longitudinal punctures.

**Male.** Antenna filiform (Fig. 12A), antennomere I much longer than others, approximate ratios of length of antennomeres I–XI 1.0: 0.5: 0.3: 0.5: 0.5: 0.5: 0.5: 0.5: 0.5: 0.5: 0.6; approximate ratios of length to width of antennomeres I–XI 4.4: 2.2: 1.9: 2.6: 2.3: 2.2: 1.9: 1.8: 1.7: 1.7: 2.6. Aedeagus (Fig. 12C, D) parallel-sided, strongly and subapically narrowed, apex tube-like and extremely small; anterior opening small, ~ 0.30 as long as aedeagus, from apical 1/13–2/5; tectum composed of one pair of sclerotized processes, large, ~ 0.76 as long as anterior opening; wide and slightly curved in lateral view; paired processes apically curved in lateral view; endophallic sclerite laterally flattened, with basal processes slightly sclerotized.

**Female.** Antenna (Fig. 12B) much smaller than in males, ratios of length of antennomeres I–XI 1.0: 0.4: 0.3: 0.3: 0.3: 0.3: 0.3: 0.4: 0.4: 0.4: 0.6; ratios of length to width of antennomeres I–XI 4.2: 1.8: 1.8: 1.6: 1.7: 1.5: 1.5: 1.7: 1.6: 1.4: 2.3. Ventrite VIII (Fig. 12E) membranous, only apical margin sclerotized, T-shaped, with two pairs of short setae at sides of apical margin, apical margin truncate, spiculum long. Spermathecal receptaculum (Fig. 12F) much longer than pump, moderately swollen; pump slightly emarginate at inner side of base; spermathecal duct with long basal part, ramus rounded. Gonocoxae (Fig. 12G) cylindrical and separated, base membranous, each gonocoxa symmetric, with dense long setae along apical and outer margin.

#### Host plant.

Unknown

#### Biology.

Unknown.

#### Etymology.

This new species is named for Jung-Chan Chen (陳榮章), the first person to collect specimens.

#### Distribution.

Only known from the type locality (Fig. 5).

### ﻿ Argopistes tsoui sp. nov.

Taxon classificationAnimaliaColeopteraChrysomelidae

BD1D0EB0-D676-5BB5-B4A0-BFB47322CF24

https://zoobank.org/7FA87DFC-55AB-45B2-AD05-A2EA64530D90

**Figure 13.**
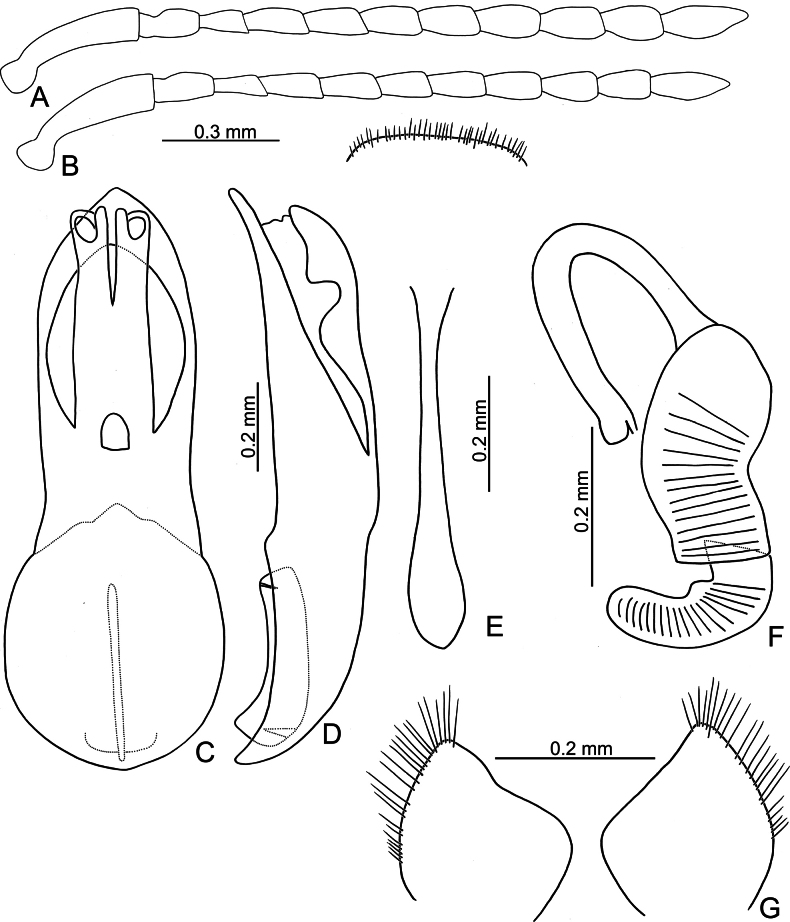
*Argopistestsoui* sp. nov. **A** antenna, male **B** antenna, female **C** aedeagus, dorsal view **D** aedeagus, lateral view **E** abdominal ventrite VIII, female **F** spermatheca **G** gonocoxae.

**Figure 14.**
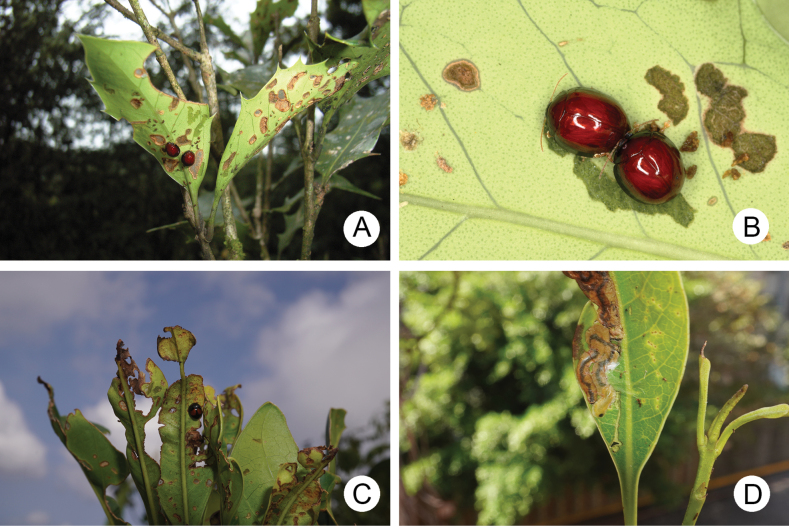
Field photographs of *Argopistes* species **A** adults of *A.tsoui* sp. nov. feeding on leaves of *Osmanthusheterophyllus***B** close-up shot of adults of *A.tsoui* sp. nov. **C** adult of *A.yuae* sp. nov. resting on underside of leaf of *Chionanthusramiflorus***D** larva mining new leaf of *C.ramiflorus*.

Figs 11C, D , 13 , 14A, B

#### Type material examined.

***Holotype*** • ♂ (TARI). Taiwan. Hsinchu: Tahunshan (大混山), 24.II.2009, leg. S.-F. Yu. • ***Paratypes*** • 1♂, 1♀ (TARI), same as holotype; • 1♀ (TARI), same but with “8.IX.2009”; Ilan: • 3♂, 3♀ (TARI), Fushan (福山) Chihwuyan (植物園 = Botanic Park), 15.II.2009, leg. M.-H. Tsou; • 4♂, 6♀ (TARI), same locality, 8.VI.2023, leg. S.-S. Lu; • 1♂ (TAFI), same but with “27.XI.2023”; • 1♀ (TAFI), same but with “28.XI.2023”; • 1♂, 1♀ (TAFI), same but with “29.XI.2023”; • 1♂, 1♀ (TARI), same but with “5.XII.2023”; • 1♀ (TARI), same but with “7.XII.2023”; • 1♀ (TARI), same but with “18.XII.2023”; • 1♂ (TARI), same but with “11.I.2024”; • 1♂ (TARI), same but with “8.III.2024”; • 4♂, 3♀ (TARI), same but with “7.V.2024”; Keelung: • 1♂, 1♀ (TARI), Hungtanshan (紅淡山), 10.V.2008, leg. M.-H. Tsou; Pingtung: • 1♀ (TARI), Lilungshan (里龍山), 5.XI.2009, leg. M.-H. Tsou; • 1♀ (TARI), Tahanshan (大漢山), 22.I.2009, leg. S.-F. Yu; • 1♀ (TARI), same locality, 20.VIII.2022, leg. Y.-T. Chung; Taoyuan: • 2♂ (TARI), Tungyanshan (東眼山), 8.VII.2007, leg. M.-H. Tsou; • 1♀ (TARI), Yongfu (永福), 24.III.2014, leg. H. Lee.

#### Diagnosis.

Adults of *A.tsoui* sp. nov. are similar to those of *A.biplagiatus* with reddish-brown elytra with wide black lateral margins, but differ from *A.biplagiatus* by the reddish-brown pronotum with wide black lateral margins (entirely black pronotum in *A.biplagiatus*). Diagnostic genitalic characters include pointed apex of aedeagus similar (Fig. 13C) to that of *A.biplagiatus* (Fig. 3C) but relatively narrower in lateral view (Fig. 13D) (relatively wider (Fig. 3D) in *A.biplagiatus*), longer, longitudinal paired sclerites of tectum (Fig. 13C) (short, curved paired sclerites of tectum (Fig. 3C) in *A.biplagiatus*), anterior opening from apical 1/10 to middle (Fig. 13C) (anterior opening from apex to apical 3/10 (Fig. 3C) in *A.biplagiatus*); triangular gonocoxae similar to those of *A.rufus* but expanding inwardly at basal 1/3 (Fig. 13G) (expanding outward at basal 1/3 (Fig. 6G) in *A.rufus*); dense setae along apical margin of abdominal ventrite VIII similar to those of *A.biplagiatus* but much denser and shorter (Fig. 13C) (less denser and longer setae on apical margin of abdominal ventrite VIII (Fig. 3E) in *A.biplagiatus*).

#### Description.

Length 3.9–4.3 mm, width 3.2–3.5 mm. Color (Fig. 11C–E) reddish brown, sides of pronotum and elytra darker, tarsi and antennae yellow. Pronotum broad, convex, lateral margin narrowly explanate; 2.2 × wider than long, disc with dense coarse punctures; lateral margin rounded, anterior margin strongly concave, posterior margin moderately convex. Elytra broadly oval, 1.0–1.1 × longer than wide, disc with dense, confused, coarse punctures.

**Male.** Antenna filiform (Fig. 13A), antennomere I much longer than others, approximate ratios of length of antennomeres I–XI 1.0: 0.4: 0.3: 0.4: 0.4: 0.4: 0.4: 0.4: 0.4: 0.4: 0.6; approximate ratios of length to width of antennomeres I–XI 4.7: 2.1: 2.2: 2.4: 2.0: 2.0: 1.9: 1.9: 1.9: 1.7: 2.7. Aedeagus (Fig. 13B, C) strongly narrowed from apical 1/5 to apex, apex pointed; anterior opening small, ~ 0.35 as long as aedeagus, from apical 1/10 to middle; tectum composed of one pair of sclerotized processes with bifurcate apices, outer apex hooked, large, ~ 1.1 as long as anterior opening; narrow and slightly curved in lateral view; endophallic sclerite laterally flattened, with small process near apex, and with basal processes membranous.

**Female.** Antenna (Fig. 13B) similar to males, ratios of length of antennomeres I–XI 1.0: 0.4: 0.3: 0.4: 0.4: 0.3: 0.4: 0.4: 0.4: 0.4: 0.5; ratios of length to width of antennomeres III–XI 4.1: 2.2: 2.4: 2.4: 2.2: 1.9: 2.1: 2.0: 1.9: 1.8: 2.9. Ventrite VIII (Fig. 13E) membranous, only apical margin sclerotized, T-shaped, with dense short setae along apical margin, spiculum long. Spermathecal receptaculum (Fig. 13F) longer than pump, moderately swollen, curved in lateral view; pump emarginate at inner side of base; spermathecal duct with elongate basal part, ramus rounded. Gonocoxae (Fig. 13G) wide and separated, base membranous, each gonocoxa asymmetric, apically narrowed from apical 1/3, with dense long setae along apical areas.

#### Host plant.

Oleaceae: *Osmanthusheterophyllus* (Fig. 13A, B), *O.kaoi* (T. S. Liu & J. C. Liao) S. Y. Lu, *O.enervius* Masam. & T. Mori, *O.fragrans*.

#### Biology.

This species seems to be univoltine. The larvae were found only during late March.

#### Etymology.

This new species is named for Mei-Hua Tsou (曹美華), the first person to collect specimens.

#### Distribution.

This new species is widespread in lowlands of Taiwan (Fig. 5).

### ﻿ Argopistes yuae sp. nov.

Taxon classificationAnimaliaColeopteraChrysomelidae

6E385736-973B-5CB0-A000-75CC7D5FD356

https://zoobank.org/D4D59AB2-7496-44F6-AD3B-C05251B4E355

**Figure 15.**
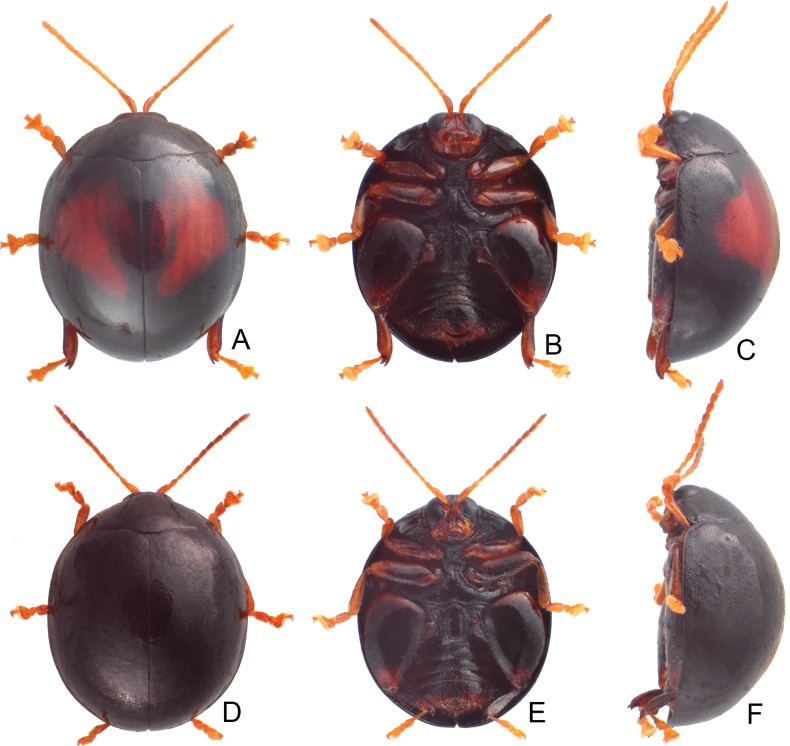
Habitus of *Argopistesyuae* sp. nov. **A** typical color form, male, dorsal view **B** ditto, ventral view **C** ditto, lateral view **D** Black color form, female, dorsal view **E** ditto, ventral view **F** ditto, lateral view.

**Figure 16.**
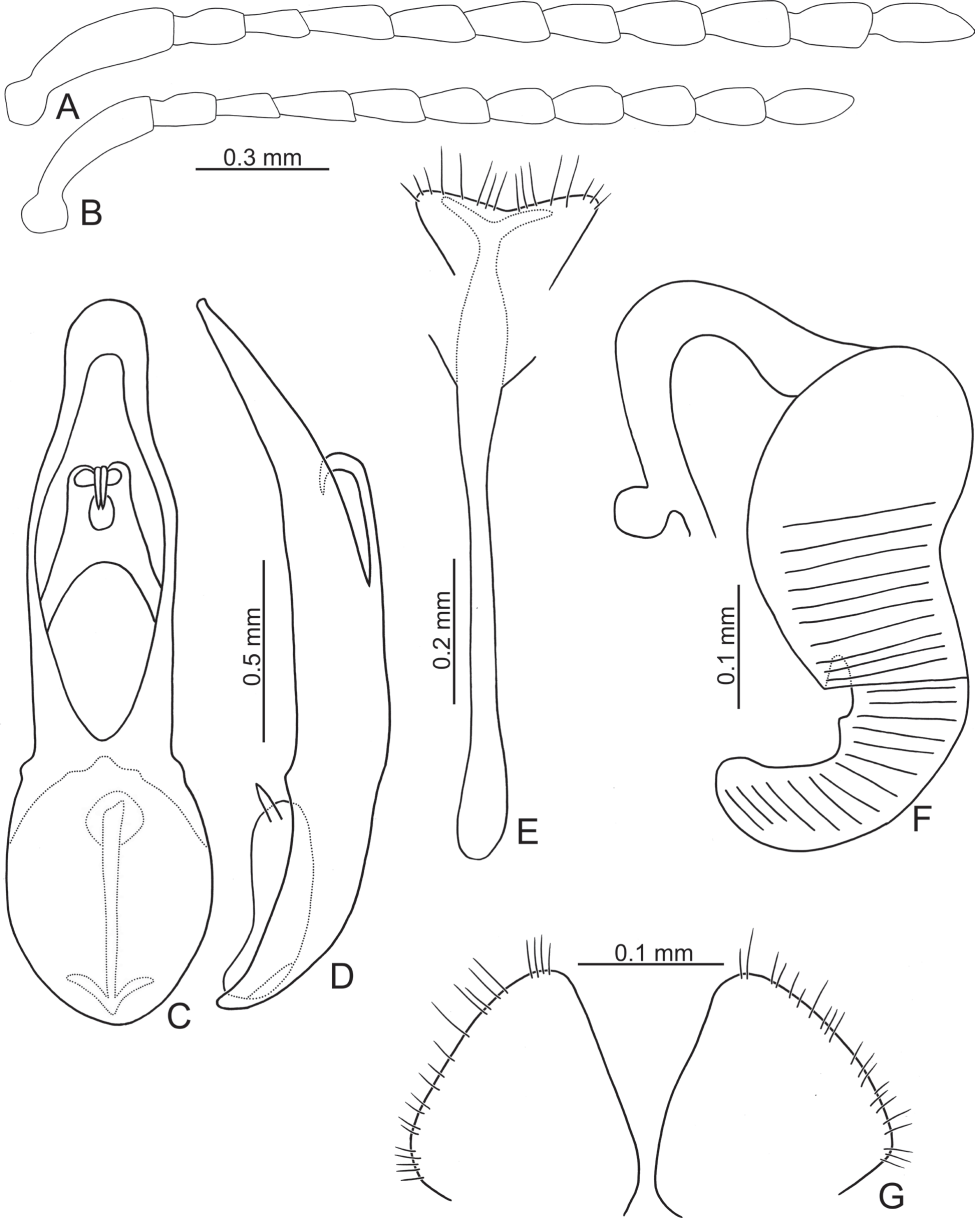
*Argopistesyuae* sp. nov. **A** antenna, male **B** antenna, female **C** aedeagus, dorsal view **D** aedeagus, lateral view **E** abdominal ventrite VIII, female **F** spermatheca **G** gonocoxae.

Figs 14C, D , 15 , 16

#### Type material examined.

***Holotype*** • ♂ (TARI). Taiwan. Taitung: Lanyu (蘭嶼), 16.IV.2023, leg. Y.-F. Hsu. ***Paratypes*** • 11♂, 7♀ (TARI), same data as holotype; • 10♂, 5♀ (TARI), same but with “20.III.2023”; • 8♂, 5♀ (TARI), same but with “17.VI.2023”; • 2♂, 3♀ (TARI), same island, 14.III.2023, leg. Y.-Y. Liu & Y.-F. Hsu; • 1♀ (TARI), same island, 28.IV.2022, leg. S-F. Yu; • 1♂ (TARI), same island, 4.IV.2016, leg. Y.-T. Chung; • 1♂ (TARI), same island, 14.IV.2013, leg. B.-X. Guo; • 1♂ (TARI), same island, 26.IV.2009, leg. U. Ong; • 3♂, 3♀ (TARI), same island, 18.III.2024, leg. Y.-F. Hsu; • 4♂, 3♀ (TARI), same island, 24.IV.2024, leg. J.-C. Chen; • 6♂, 3♀ (NHMUK), same island, Lanyu Weather Station (蘭嶼氣象站), 22°02.238'N, 121°33.287'E, 26.VII.2008, hand collecting, leg. M. V. L. Barclay & H. Mendel; • 2♂, 1♀ (TARI), same island, Tataienchih (大天池), 19.III.2024, leg. Y.-F. Hsu.

#### Diagnosis.

Adults of this new species are not separable from those of *A.rufus* except by genitalic characters, including parallel-sided apex of aedeagus with anterior opening very close to apex of aedeagus, from apical 1/12–3/5 (Fig. 16C) (apically narrowed aedeagus with anterior opening not so close to apex of aedeagus, from apical 1/8–3/5 (Fig. 6C) in *A.rufus*); deeply notched apical margin of abdominal ventrite VIII (Fig. 16E) in females (shallowly notched apical margin of abdominal ventrite VIII (Fig. 6E) in females of *A.rufus*). In addition, this new species is restricted to Lanyu Island, and thus is isolated from other species geographically. Moreover, larvae and adults of this new species feed on leaves of *Chionanthusramiflorus* Roxb. (Fig. 14C, D) but not those of *Osmanthusfragrans* based on laboratory rearing tests. Thus, both species are allopatric ecologically since *Osmanthusfragrans* is one of the host plants for *A.rufus*.

#### Description.

Length 4.2–4.3 mm, width 3.5 mm. Color (Fig. 15A–C) blackish brown, elytron with one large transparent area at basal 1/3, near or connected with suture; tarsi and antennae yellowish brown. Pronotum broad, convex, lateral margin narrowly explanate; 2.3 × wider than long, disc with dense, coarse punctures; lateral margin rounded, anterior margin strongly concave, posterior margin moderately convex. Elytra broadly oval, 1.1 × longer than wide, disc with confused, dense, fine punctures.

**Male.** Antenna filiform (Fig. 16A), antennomere I much longer than others, approximate ratios of length of antennomeres I–XI 1.0: 0.4: 0.3: 0.5: 0.4: 0.4: 0.4: 0.5: 0.5: 0.4: 0.6; approximate ratios of length to width of antennomeres I–XI 3.8: 2.0: 2.0: 2.7: 2.0: 1.9: 1.9: 1.9: 1.8: 1.9: 2.8. Aedeagus (Fig. 16C, D) parallel-sided from basal 1/3–2/3, apically narrowed from apical 1/3–1/6, apex tube-like; anterior opening large, ~ 0.53 as long as aedeagus, from apical 1/12–3/5; tectum composed of one pair of sclerotized processes, small, ~ 0.43 as long as anterior opening; wide and slightly curved in lateral view; paired processes curved at apical 1/3 in lateral view; endophallic sclerite laterally flattened, with basal processes slightly sclerotized, and one pair of small processes near apex.

**Female.** Antenna (Fig. 16B) similar to males, ratios of length of antennomeres I–XI 1.0: 0.4: 0.3: 0.4: 0.4: 0.4: 0.4: 0.4: 0.4: 0.4: 0.5; ratios of length to width of antennomeres III–XI 4.9: 1.9: 2.3: 2.5: 2.0: 1.8: 1.9: 1.8: 1.8: 1.9: 2.5. Ventrite VIII (Fig. 15E) weakly sclerotized, T-shaped, with several pairs of setae along apical margin, setae smaller at sides, apical margin medially depressed, spiculum long. Spermathecal receptaculum (Fig. 16F) longer than pump, moderately swollen, curved in lateral view; pump emarginate at inner side of base; spermathecal duct with long basal part, ramus rounded. Gonocoxae (Fig. 16G) wide and separated, base membranous, each gonocoxa asymmetric, apically narrowed from near base, with sparse setae along apical areas, setae longer at apical 1/2.

#### Variation.

A few specimens have black bodies and lack red spots on elytra (Fig. 14D–F).

#### Host plant.

Oleaceae: *Chionanthusramiflorus* Roxb.

#### Biology.

This species seems to be univoltine. The larvae (Fig. 14D) were found only during late March.

#### Etymology.

This new species is named for Su-Fang Yu (余素芳), the first person to collect specimens.

#### Distribution.

Endemic to Lanyu Island (Fig. 5).

### ﻿Key to Taiwanese species of *Argopistes*

Kimoto (1965) provided a key to Japanese species of *Argopistes*. We think that the key is appropriate for Japan but not other countries due to color variations or/and color patterns for species elsewhere. A key to Taiwanese species of *Argopistes* is provide as follows:

**Table:** 

1	General color yellowish brown, elytra with black spots; aedeagus (Fig. 7C, D) apically narrowed, and apex widely rounded, apical margin of anterior opening far from (apical 1/8) apex of male aedeagus	***A.rufus* Chen**
–	General color black or reddish brown	**2**
2	General color reddish brown, with black, wide margins along pronotum and elytra; aedeagus (Fig. 13B, C) strongly narrowed from apical 1/5 to apex, apex pointed; anterior opening from apical 1/10 to middle; narrow and slightly curved in lateral view	***A.tsoui* sp. nov.**
–	General color black, with or without yellowish brown margins along pronotum and elytra	**3**
3	Elytra with one pair of red spots, but lacking yellowish brown margins along pronotum and elytra	**4**
–	Elytra without red spots, and with yellowish-brown margins on pronotum and elytra; aedeagus (Fig. 12C, D) parallel-sided, strongly subapically narrowed, apex tube-like and extremely small; anterior opening from apical 1/13–2/5; wide and slightly curved in lateral view	***A.jungchani* sp. nov.**
4	Only coarse punctures on elytra, wider interspace between eyes; aedeagus (Fig. 3C, D) apically and strongly narrowed from apical 1/5, apex pointed; anterior opening very small, from apex to apical 3/10; extremely wide in lateral view	***A.biplagiatus* Motschulsky**
–	Coarse and fine punctures confused on elytra, narrower interspace between eyes	**5**
5	Aedeagus (Fig. 7C, D) apically narrowed, apex widely rounded, apical margin of anterior opening far from apex of male aedeagus (apical 1/8)	***A.rufus* Chen**
–	Aedeagus (Fig. 16C, D) apically narrowed, and apex parallel-sided, apex widely rounded, apical margin of anterior opening close to apex of male aedeagus (apical 1/12)	***A.yuae* sp. nov.**

## ﻿Discussion

Some Taiwanese species can be identified by their characteristic color patterns, including *A.tsoui* sp. nov., *A.jungchani* sp. nov., and the yellowish-brown form of *A.rufus* Chen. Male aedeagi are more diagnostic. Genital characters in females such as abdominal ventrite VIII, spermatheca, and gonocoxae are more or less diagnostic, but combinations of these morphological characters and biological information can form the basis for a sound taxonomy of the genus.

Although members of *Argopistes* are oligophagous or monophagous on Oleaceae, only *A.rufus* has established populations in small islands surrounding Japan, China, and Taiwan. Five islands (Beigan Island 北竿島, Nangan Island 南竿島, Dongju Island 東莒島, Xiju Island 西莒島, Dongyin Island 東引島) of the Matsu Islands were investigated during the spring 2024 field season. Populations of *A.rufus* were found on only Beigan Island and Nangan Island feeding on *Chionanthusretusus* and *Ligustrumjaponicum*. *Chionanthusretusus* and *Ligustrumjaponicum* were transported to both islands, the largest of the Matsu Islands, as ornamental trees. This supports the idea that *A.rufus* can be an invasive insect pest, invading islands as a result of exportation of ornamental trees of Oleaceae. In addition, populations of *A.rufus* expanded dramatically in Kinmen Island (金門島). One population was found at one locality of Jinsha township (金沙鎮) in the middle of April 2023 and one adult was collected at another locality (Botanical Park) at Jinhu township (金湖鎮) in the summer (July) of 2023. We have now found larvae attacking new leaves of *Osmanthusfragrans* at a guesthouse in Jinhu township (金湖鎮), in late May 2024.

## Supplementary Material

XML Treatment for Argopistes biplagiatus

XML Treatment for Argopistes rufus

XML Treatment for Argopistes tsekooni

XML Treatment for Argopistes unicolor

XML Treatment for Argopistes jungchani

XML Treatment for Argopistes tsoui

XML Treatment for Argopistes yuae

## ﻿Additional information

### Author ORCIDs

Chi-Feng Lee https://orcid.org/0000-0003-1996-0557

Ming-Yao Chiang https://orcid.org/0000-0002-1514-7086

### Data availability

All of the data that support the findings of this study are available in the main text.
